# Revealing Physical Mechanisms of Spatial Pattern Formation and Switching in Ecosystems via Nonequilibrium Landscape and Flux

**DOI:** 10.1002/advs.202501776

**Published:** 2025-04-25

**Authors:** Jie Su, Wei Wu, Denis D. Patterson, Simon Asher Levin, Jin Wang

**Affiliations:** ^1^ Center for Theoretical Interdisciplinary Sciences Wenzhou Institute University of Chinese Academy of Sciences Wenzhou 325001 China; ^2^ High Meadows Environmental Institute Princeton University Princeton NJ 08544 USA; ^3^ Department of Ecology and Evolutionary Biology Princeton University Princeton NJ 08544 USA; ^4^ Department of Mathematical Sciences Durham University Durham DH13LE UK; ^5^ Department of Chemistry and of Physics and Astronomy State University of New York of Stony Brook Stony Brook New York 11794 USA

**Keywords:** critical transition, landscape‐flux theory, mode expansion method, spatial pattern, semi‐arid ecosystem

## Abstract

Spatial patterns are widely observed in numerous nonequilibrium natural systems, often undergoing complex transitions and bifurcations, thereby exhibiting significant importance in many physical and biological systems such as embryonic development, ecosystem desertification, and turbulence. However, how spatial pattern formation emerges and how the spatial pattern switches are not fully understood. Here, a landscape‐flux field theory is developed using the spatial mode expansion method to uncover the underlying physical mechanism of the pattern formation and switching. The landscape and flux field are identified as the driving force for spatial dynamics and applied this theory to the critical transitions between spatial vegetation patterns in semi‐arid ecosystems, revealing that the nonequilibrium flux drives the switchings of spatial patterns. The emergence of pattern switching is revealed through the optimal pathways and how fast this occurs via the speed of pattern switching. Furthermore, both the averaged flux and the entropy production rate exhibit peaks near pattern switching boundaries, revealing dynamical and thermodynamical origins for pattern transitions, and further offering early warning signals for anticipating spatial pattern switching. This work thus reveals physical mechanisms on spatial pattern‐switching in semi‐arid ecosystems and, more generally, introduces a useful approach for quantifying spatial pattern switching in nonequilibrium systems, which further offers practical applications such as early warning signals for critical transitions of spatial patterns.

## Graphical abstract

The universality of the landscape‐flux theory with the mode expansion method for analyzing spatial patterns is illustrated by applying it to vegetation patterns in semi‐arid ecosystems. The framework quantifies global dynamics and most probable transition pathways between ecosystem states under random forcing. This methodology offers a universal approach to characterizing the evolution of spatial patterns in nonequilibrium systems.

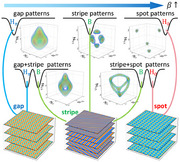

## 1 Introduction

Spatial patterns are widely observed in numerous nonequilibrium systems across a range of scientific fields, prominent examples include Turing patterns in chemical reactions,^[^ ^1^, ^2^, ^3^ ^]^ pattern formation during embryonic development,^[^ ^4^, ^5^, ^6^, ^7^ ^]^ patterns in vegetation or animal distributions in ecological models,^[^ ^8^, ^9^, ^10^, ^11^, ^12^, ^13^, ^14^, ^15^, ^16^, ^17^, ^18^, ^19^, ^20^ ^]^ and complex spatial patterns emerging in turbulence.^[^ ^21^, ^22^ ^]^ These various spatial patterns have attracted considerable interest for their complex emergence and evolution.^[^ ^23^, ^24^, ^25^, ^26^, ^27^ ^]^ For instance, the complex ecosystems and Earth system components can evade tipping points through the spatial pattern formation, such as Turing patterns and coexistence state.^[^ ^23^ ^]^ Specific spatial patterns of proliferation and necrosis can explain clonal expansion, emergence of parallel evolution, and microdiversity in tumours.^[^ ^24^ ^]^ Despite these significant progresses, how the spatial patterns form and switch are not fully understood. Such an understanding requires global description. Here, we developed a landscape and flux field theory via spatial mode expansion method to reveal the physical mechanism of pattern formation and switching. The landscape and flux theory for well mixed spatially homogeneous systems identified the driving forces as landscape gradient/flux and has been successful in investigating global information as well as the dynamical and thermodynamical mechanisms of nonequilibrium systems involving the cell cycle, cell differentiation and development, cancer, and ecological systems.^[^ ^28^, ^29^, ^30^, ^31^, ^32^, ^33^, ^34^, ^35^, ^36^, ^37^, ^38^ ^]^ We need to generalize this theory to spatially extended systems.^[^ ^39^, ^40^, ^41^, ^42^ ^]^ However, exact solutions for nonlinear systems remain almost impossible due to the complex nature of spatially dependent fields involving huge numbers of degrees of freedom (DOFs) in the spatial locations. Therefore, it is of paramount importance to find a suitable method to describe the global dynamics and evolution of patterns in spatial nonequilibrium systems.

An effective approach to address this problem is to apply the mode expansion method from field theory,^[^ ^43^, ^44^, ^45^ ^]^ which maps numerous DOFs of spatial locations onto certain representative spatial modes. This mapping can facilitate the transformation of the complex functional probabilistic evolution equation into a simpler Fokker‐Planck equation in the mode space. By developing the landscape and flux theory in this mode space, two key benefits for investigating spatial patterns can then be realized. First, we can quantify the potential landscape in the mode space, which reflects the global stability of the spatial pattern systems; such as locations and weights (potential depths) of steady states. Second, we can obtain the flux field and the entropy production rate (EPR), which can be used to explore the nonequilibrium dynamical and thermodynamical mechanisms of the emergence and switching of spatial patterns. Therefore, introducing the landscape and flux theory with the mode expansion method to the study of spatially patterned nonequilibrium systems has significant potential to generate deeper physical insights into pattern dynamics and possible practical applications, such as quantifying the likelihood of regime shifts and thereby providing early warning signals.

In this paper, we report a practical example to highlight the potential of applying the nonequilibrium landscape and flux theory combined with the mode expansion method (with appropriate truncations) to spatially extended systems. The model we study concerns spatial vegetation patterns located in semi‐arid ecosystem,^[^ ^46^, ^47^, ^48^ ^]^ which are widely distributed in many regions including parts of Africa,^[^ ^49^, ^50^, ^51^, ^52^ ^]^ Australia,^[^ ^53^, ^54^ ^]^ and Mexico.^[^ ^55^ ^]^ The vegetation exhibits diverse spatial patterns, such as stripe, gap, spot^[^ ^46^, ^47^, ^48^, ^49^, ^56^ ^]^ and irregular mosaics.^[^ ^51^, ^52^ ^]^ It is noteworthy that these spatial patterns may provide an indication of ecosystem resilience, for instance, spot patterns have been shown to be a potential early warning signal of desertification.^[^ ^47^, ^57^, ^58^, ^59^, ^60^ ^]^ Beyond common factors such as the rainfall, the positive feedback regulation between the plant biomass and water also plays a significant role in generating different types of spatial vegetation patterns located in a semi‐arid ecosystem.^[^ ^47^, ^48^, ^61^, ^62^, ^63^ ^]^ That is, as the feedback intensity increases, the vegetation pattern switches from gap to stripe and ultimately to spot pattern.^[^ ^48^ ^]^

In the current study, we begin with stochastic evolution equations for the vegetation biomass and water fields that are then transformed into reduced stochastic Langevin dynamic equations and a Fokker‐Planck equation based on several representative spatial modes by using the mode expansion method with appropriate truncations. Subsequently, by applying the landscape and flux theory in the mode space, we quantify the potential landscape of the semi‐arid ecosystems which provides a global picture of the dynamics. We observe that as the soil‐water diffusion feedback intensity increases, the landscape transforms successively from gap to gap/stripe, stripe, stripe/spot, and finally spot states. Besides, we find the nonequilibrium flux inside the landscape often acts against the potential gradient, providing a driving force for the system to switch from one spatial pattern to another one. Detailed analysis reveals that the non‐overlapping transition paths between alternative stable states, reflecting the time‐reversal symmetry breaking of the system, is attributed to the combined effect of the potential gradient force, the flux force and the spatially dependent noise force. Moreover, the switching time between states corresponds to the barrier height as well as the closest basin distance. Both the transition paths and the switching time offer new insights into the physical mechanisms of switching between spatial patterns. More interestingly, both the averaged flux and EPR present peaks near the phase boundaries, not only revealing the dynamical and thermodynamical mechanisms of the critical transition, but also providing early warning signals for the desertification. Furthermore, additional simulations explore the influence of ecosystem size (noise) and other parameters, demonstrating the generality and robustness of our method.

## 2 Results and Discussion

### 2.1 The Global Stability of Semi‐Arid Ecosystems

**Figure 1:**
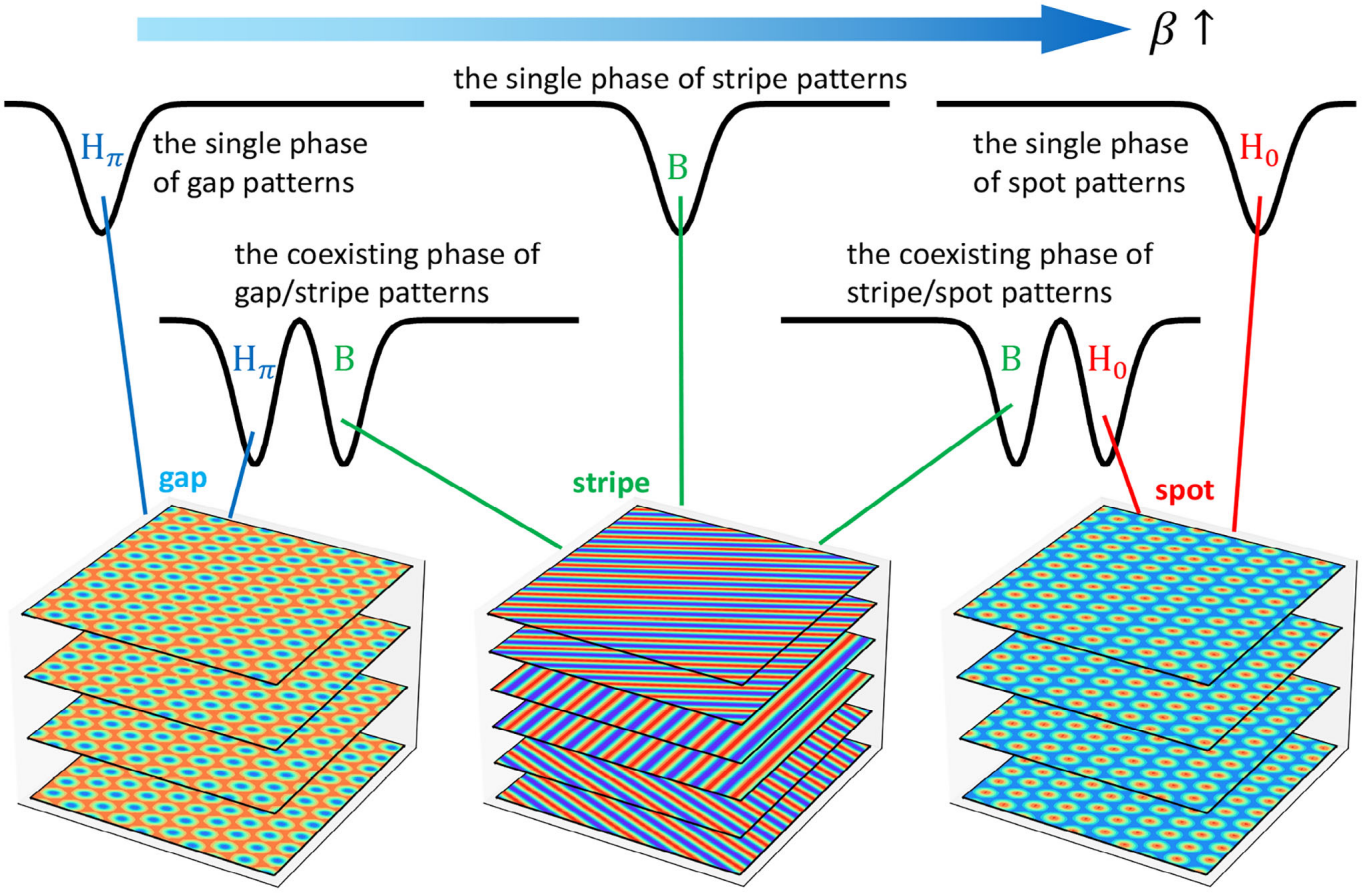
The conceptual picture of the potential landscape depending on β. As β increases, the landscape undergoes transitions: from a single potential landscape basin in *H*_π_ (gap pattern) state to two basins in both *H*_π_ and *B* states, then to a single basin in *B* (stripe pattern) state, followed by two basins in both *B* and *H*_0_ states and finally to a single basin in *H*_0_ (spot pattern) state. Due to different phase shifts or orientations among spatial patterns, the number of *H*_π_, *B* and *H*_0_ states are 4, 6 and 4, respectively.

First, we present the conceptual picture of the quantified potential landscape depending on the soil‐water diffusion feedback intensity β, as illustrated in **Figure** **1** (The detailed models and methods can be found in Experimental Section and the Supporting Information). As β increases, the schematic diagram of the landscape in the mode space shifts from a single potential basin in *H*_π_ (gap pattern) state to two basins in both *H*_π_ and *B* states, then to a single basin in *B* (stripe pattern) state, followed by two basins in both *B* and *H*_0_ states and finally to a single basin in *H*_0_ (spot pattern) state. *H*_π_, *B*, and *H*_0_ states respectively have *four*, *six*, and *four* steady states located at different regions in the mode space (see details in Supporting Information and Figure S4, Supporting Information). These steady states are degenerate with equal probability or potential on the landscape, and patterns located in them all have the same shape but exhibit different phase shifts or orientations between them (see detailed description in Supporting Information and Figure S5–S8, Supporting Information). We will also discuss the degenerate steady states of gap, stripe and spot patterns and their relationships with the positions (amplitudes) of key modes in the Discussion and Conclusion section later.

**Figure 2:**
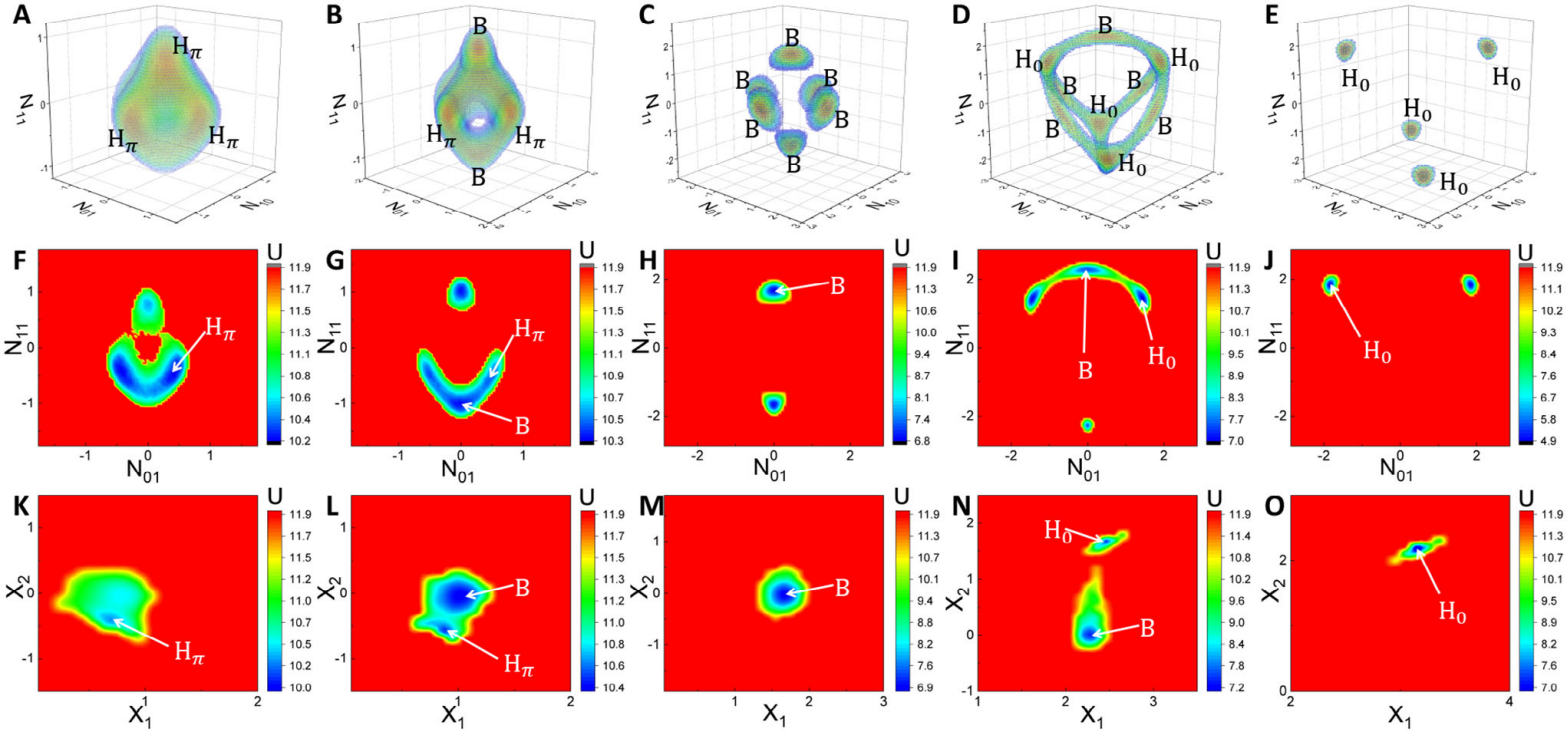
The typical potential landscape of the semi‐arid ecosystem in the mode space. Landscape for gap(*H*_π_), gap(*H*_π_)/stripe(*B*), stripe(*B*), stripe(*B*)/spot(*H*_0_) and spot(*H*_0_) phases in the 3D mode space (A–E), the sections of *N*_01_ − *N*_10_ = 0 (F–J), and the *X*_1_‐*X*_2_ mapping space (K–O). The parameters are β = 0.003 for (A,F,K), 0.004 for (B,G,L), 0.009 for (C,H,M), 0.018 for (D,I,N), and 0.026 for (E,J,O). The color in (A–E) changes from blue to red representing that the potential changes from high to low.

Then simulations of stochastic Langevin equations (Equation (7) in Experimental Section) are performed and the potential landscape based on the mode space can be calculated by Equation (8) in Experimental Section. The obtained 3‐D landscape of typical patterns are illustrated in **Figure** **2**A–E; 2‐D sections of the plane *N*_01_ − *N*_10_ = 0 are presented in Figure 2F–J. For β equal to 0.003, 0.004, 0.009, 0.018, and 0.026, the potential landscape in the section of *N*_01_ − *N*_10_ = 0 presents pairs of potential basins located in *H*_π_ state (Figure 2F), *H*_π_+*B* states (Figure 2G), *B* state (Figure 2H), *B*+*H*_0_ states (Figure 2I) and *H*_0_ state (Figure 2J), respectively, corresponding to the five landscapes shown in the conceptual picture in Figure 1. Furthermore, we introduce a mapping method to merge those degenerate potential basins that are located in the same state together. We fix $X_{1}=\sqrt{N_{01}^{2}+N_{10}^{2}+N_{11}^{2}}$, *X*_2_ = sgn(*N*_01_ *N*_10_ *N*_11_)(|*N*_01_| + |*N*_10_| + |*N*_11_| − *X*_1_) with sgn(*x*) the sign function, and then map the 3‐D mode‐space landscape into the *X*_1_‐*X*_2_ phase space. On this basis, potential basins in *H*_π_, *B* and *H*_0_ states will be mapped into the basin located in the region with *X*_1_ > 0, *X*_2_ < 0; *X*_1_ > 0, *X*_2_ = 0; and *X*_1_ > 0, *X*_2_ > 0; respectively. As shown in Figure 2K–O, the potential basins are quantified and illustrated through the locations in gap, gap/stripe, stripe, stripe/spot and spot states, as β increases.

**Figure 3:**
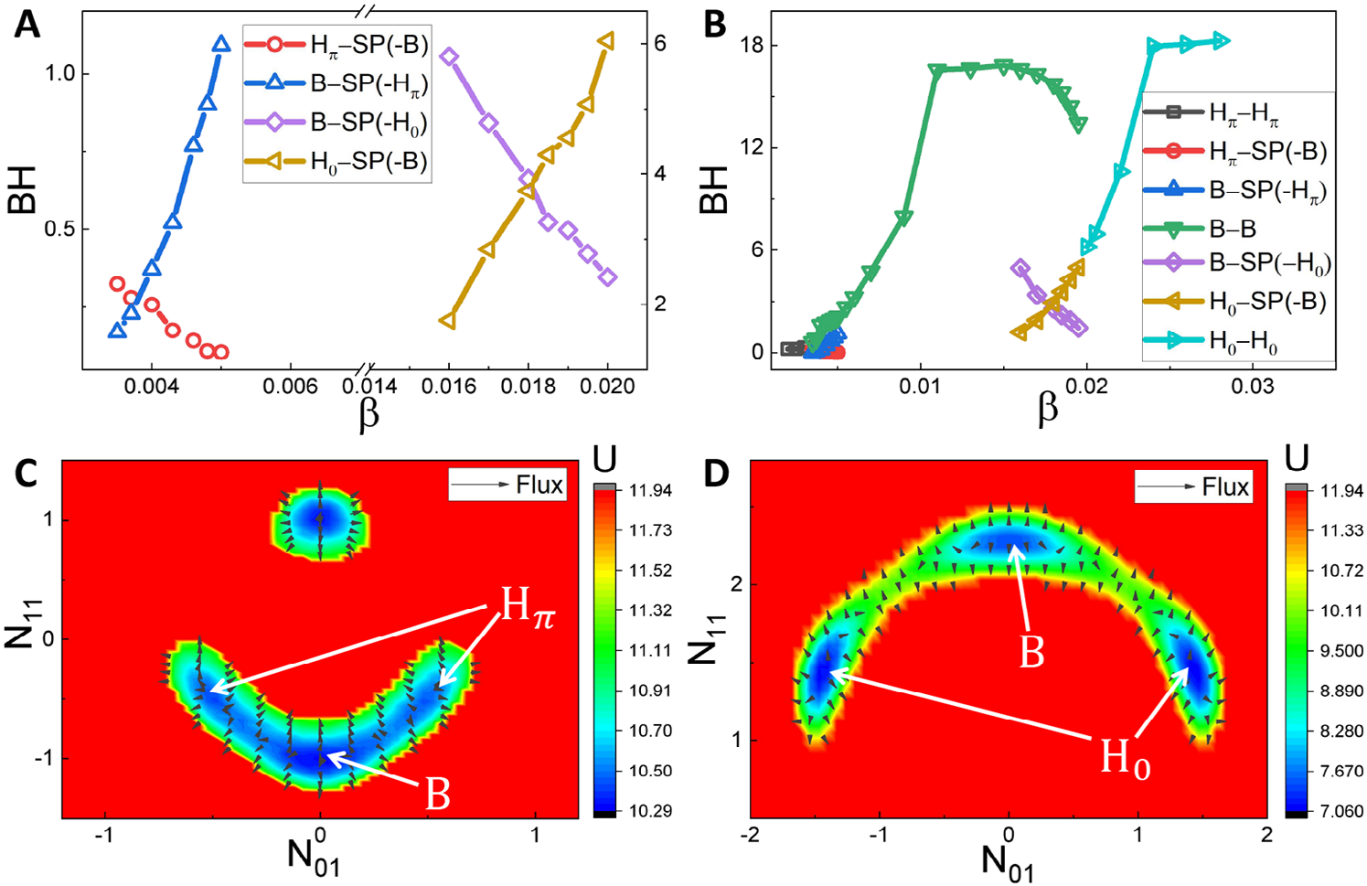
The barrier height and the flux field of the semi‐arid ecosystem. A) Dependence of the inter‐state barrier height on β in the coexisting phases. B) Dependence of the barrier height on β, including both the inner‐state and inter‐state barriers. C,D) The potential section of *N*_01_ − *N*_10_ = 0 (colored background) and flux field (black arrows) of the ecosystem in the coexisting phases with (C) β = 0.004 and (D) β = 0.018. *H*_π_, *B*, and *H*_0_ states are the gap, stripe and spot pattern, respectively.

Based on the landscape, global topography such as the barrier height can be obtained. As shown in **Figure** **3**A, we exhibit the inter‐state barrier height (IntBH) in the *X*_1_ − *X*_2_ phase space and observe two different types of barriers in the coexisting phases. Interestingly, for the gap/stripe coexisting phase, the IntBH between potential basins in the gap state and the saddle point decreases as β increases, while the one in the stripe state increases, indicating that the weight of gap and stripe states become lighter and heavier, respectively. Similarly, for the stripe/spot coexisting phase, the IntBH between the basin in the former(latter) state and the saddle point decreases(increases) as β increases, implying that the weight of the former(latter) state becomes lighter and lighter(heavier and heavier). In addition to the inter‐state barrier between potential basins located in different states, the inner‐state barrier between the closest basins situated in the same state can be presented through the landscape in the mode space. As shown in Figure 3B, we observe that the inner‐state barrier height (InnBH) in the single phase increases as a whole with the rise of β. Therefore, the barrier height can be used as a global stability measure quantifying the capability of spatial pattern switching. Higher barrier implies harder for state to switch and thus global stability, while lower barrier indicates higher chances of spatial pattern switching and thus lower stability. Since the spot vegetation pattern implies the desertification precursor, the barrier height of the spot spatial pattern then becomes a corresponding early warning for the environmental protection.

Next, we focus on the nonequilibrium flux from Equation (9) in Experimental Section. The obtained flux field for typical patterns in the 3‐D mode space are shown in Figure S9 (Supporting Information), which is also challenging to see clearly, similar to the potential landscape in the 3‐D mode space. Similarly, we choose the section of the plane *N*_01_ − *N*_10_ = 0 to present the flux field more intuitively. As examples in the coexisting phases, two typical landscapes with flux for β = 0.004 and β = 0.018 are shown in Figure 3C,D, respectively. The flux typically points to the opposite direction of the potential gradient, acting as a driving force for the system to switch from one state to the other one. This means that the vegetation pattern can switch more easily between the two pattern states in the coexisting phase with the help of the nonequilibrium flux.

### 2.2 The Transition Paths and Rate Between Different Spatial Patterns

**Figure 4:**
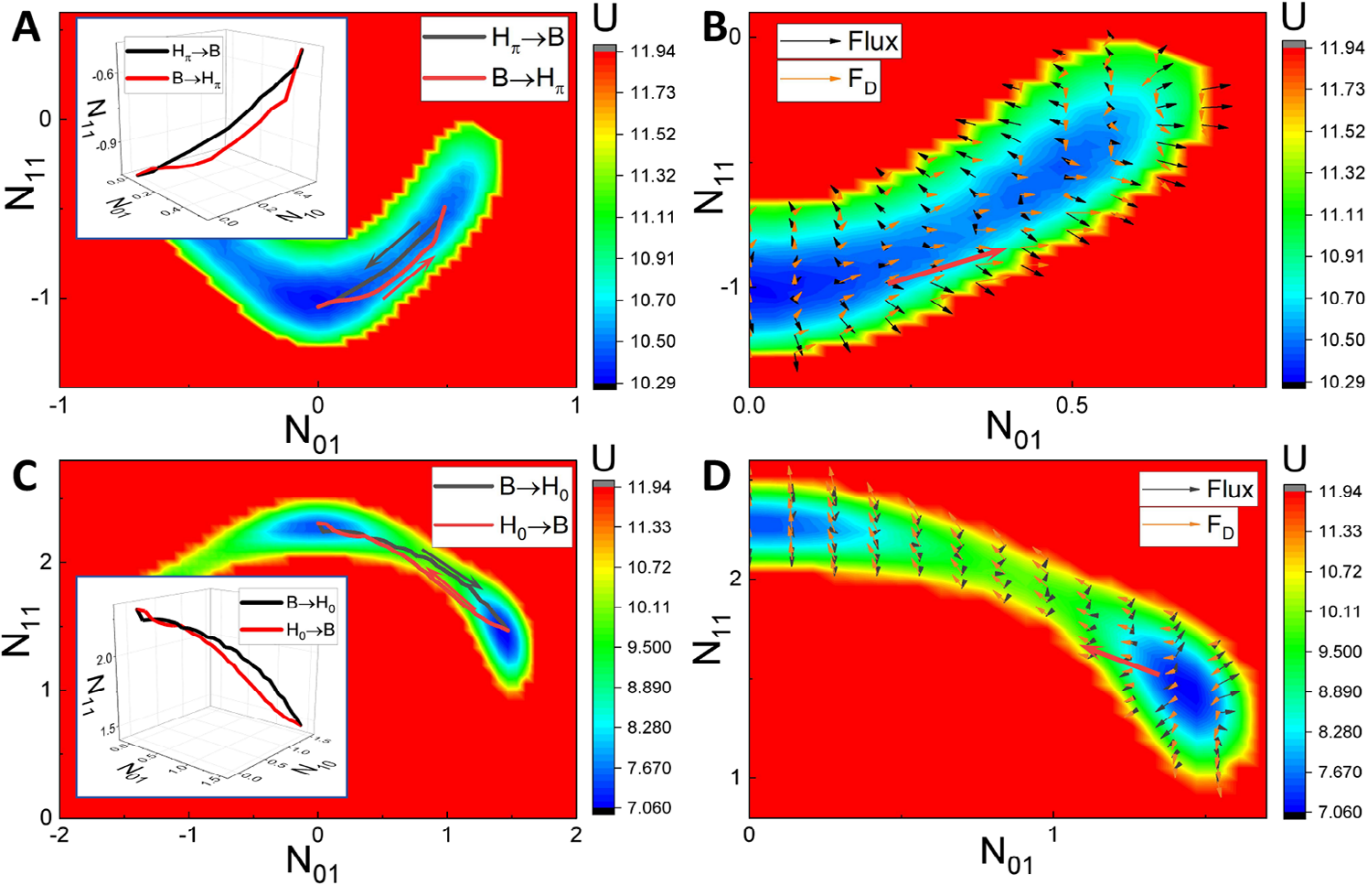
The transition paths between different states in the coexisting phase. A) The transition paths between the gap (*H*_π_) and stripe (*B*) states in the potential section of *N*_01_ − *N*_10_ = 0 for β = 0.004. B) The zoomed image around the transition paths in A) consisting of the potential (colored background), flux (black arrows) and the spatial dependent noise focre (orange arrows). C) The transition paths between the spot (*H*_0_) and stripe (*B*) states in the potential section of *N*_01_ − *N*_10_ = 0 for β = 0.018. D) The zoomed image around the transition paths in (C). The insets in (A) and (C) are the transition paths in the 3D mode space.

In addition to the global information obtained by establishing the landscape and flux field in the mode space, we can also explore how the spatial pattern switching process is realized and how fast this occurs. A pair of transition paths between the gap state (located in the region with *N*_01_ = *N*_10_ = −*N*_11_ > 0) and the stripe state (located in the region with *N*_01_ = *N*_10_ = 0 and *N*_11_ < 0) for β = 0.004 in the gap/stripe coexisting phase is shown in **Figure** **4**A by using the dominant path approach.^[^ ^64^ ^]^ We can observe that the transition path from the gap state to the stripe state (the black line) does not overlap with its inverse path from the stripe state to the gap state (the red line), implying that the time‐reversal symmetry is broken in such situations. To explain the non‐overlap of this pair of transition paths, a zoomed image around these paths is presented in Figure 4B. It is found that the transition path from the gap state to the stripe state (the black line in Figure 4A) seems to move along with the lowest potential, which is easily and naturally to be understood. Interestingly, the transition path from the stripe state to the gap state (the red line in Figure 4A) shifts slightly to the right of the black path, consistent with the combined effect including the potential gradient force, the flux force breaking the detailed balance of the equilibrium system and representing the net flow in or out of the system (black arrows in Figure 4B), as well as the force related to the spatial dependent noise (orange arrows). For equilibrium systems, the forward and backward paths follow the same route so that these gradient‐driven paths overlap with each other and the system is time‐reversal. However, when the system becomes nonequilibrium, the curl flux that prefers global movement due to the rotational nature of this nonequilibrium driving force, resulting in that the forward and backward paths follow different routes. Consequently, this pair of transition paths becomes non‐overlapping and the time‐reversal symmetry of the system is broken.

Similarly, for the situation in the stripe/spot coexisting phase at β = 0.018 (Figure 4C), we find that the transition path from the stripe state to the spot state (the black line) moves along with the lowest potential, while the one from the spot state to the stripe state (the red line) shifts slightly to the left of the black path. This can also be explained by the combined effect consisting of the potential gradient force, the flux force and the force related to the spatial dependent noise (Figure 4D). Additionally, the pair of transition paths at β = 0.018 overlap less than those in the case β = 0.004, indicating that the degree of the time‐reversal symmetry breaking in the stripe/spot coexisting phase is greater than that in the gap/stripe coexisting phase. Moreover, we focus on the dominant modes on this transition path (the black line in Figure 4C) at β = 0.018 during the nucleation process (discussed in the next section). We find that *N*_01_ and *N*_10_ play dominant roles on this path. This indicates that when the ecosystem changes from the stripe pattern to the spot one, it tends to increase the maximum magnitude of vegetation biomass *n* first (before the nucleation formation) and then change the pattern shape later (see details in Supporting Information and Figures S10 and S11, Supporting Information). In other words, the emergence of the non‐uniform magnitude of vegetation biomass in the stripe pattern predicts the pattern switching to a spot shape, which may herald the onset of desertification.

**Figure 5:**
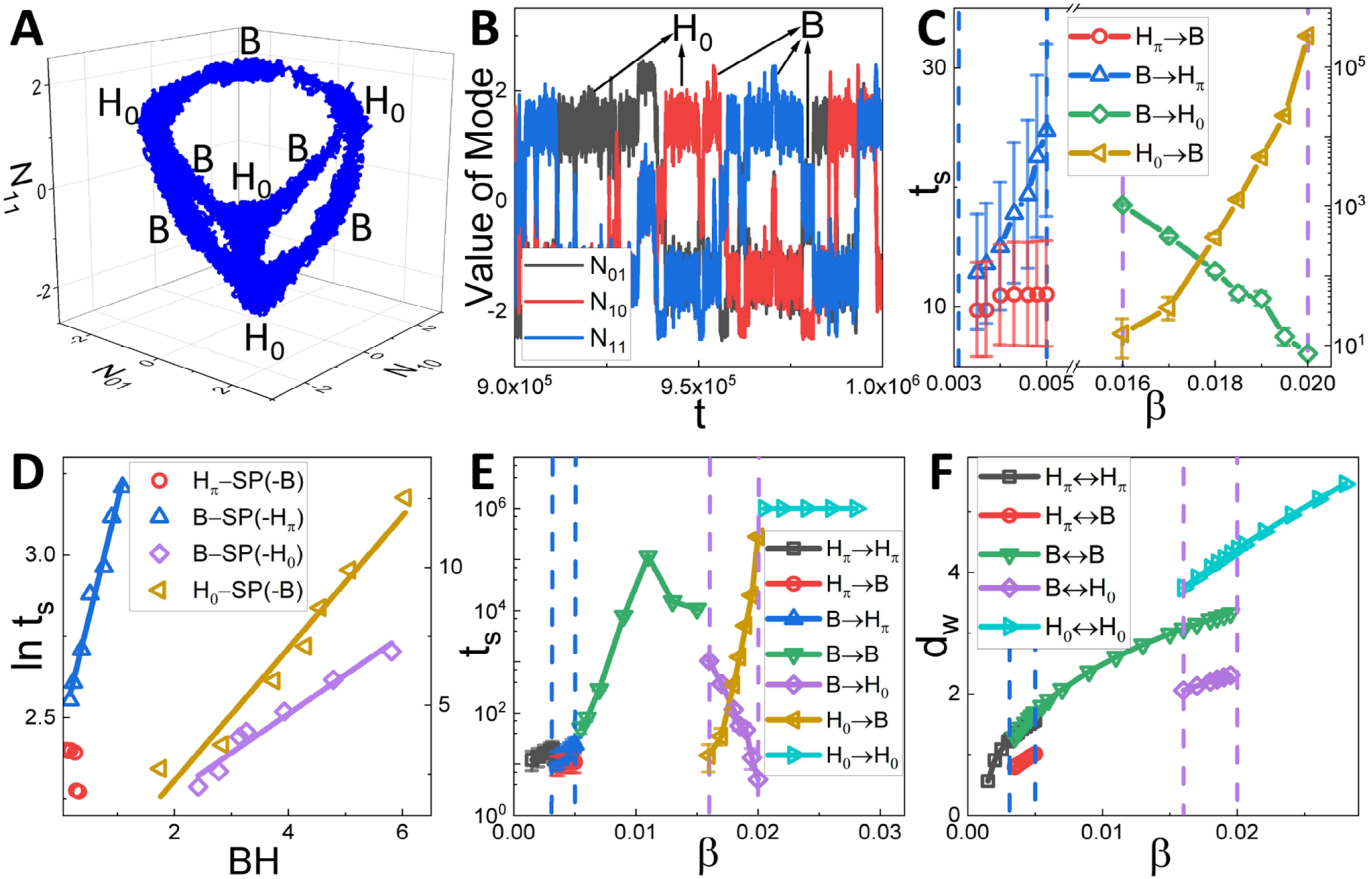
The transition rate between different states. A) Trajectory of the semi‐arid ecosystem with β = 0.018 in the steady state. B) Time series of *N*_01_, *N*_10_ and *N*_11_ in (A). C) Dependence of the inter‐state switching time *t*_*s*_ on β in the coexisting phases. D) ln *t*_*s*_ as functions of the inter‐state barrier height. E) Dependence of *t*_*s*_ on β, including both the inter‐state and inner‐state switching time. F) Dependence of *d*_*w*_ on β. The blue and purple dashed lines in (C,E,F) denote the phase boundaries.

Simultaneously, the transition rate can be understood by investigating the switching time from one state to the another. We perform an additional simulation for one ecosystem evolving in the mode space after reaching the steady state. As an example illustrated in **Figure** **5**A,B, we present the trajectory in 3D mode space and the time series of *N*_01_, *N*_10_, and *N*_11_ at β = 0.018. The time before the system jumps from one state to another is referred to as the single switching time. By performing time averaging, the obtained inter‐state switching time *t*_*s*_ dependent on β in the coexisting phases are shown in Figure 5C. For the stripe/spot coexisting phase, *t*_*s*_ from the potential basin in the stripe(spot) state to the one in the spot(stripe) state decreases(increases), as β increases. All these results are aligned with the behaviors of the IntBH (Figure 3A) and can be similarly understood through it. In other words, the switching time between the states is exponentially related to the barrier height in between. As shown in Figure 5D, the quantity ln *t*_*s*_ increases linearly as the barrier height increases in the stripe/spot coexisting phase (purple and yellow symbols and lines). Therefore, the speed of spatial pattern switching is strongly related to the barrier height between these states.

However, for the gap/stripe coexisting phase (Figure 5C), both of *t*_*s*_s increase as β increases. As illustrated in Figure 5D, ln *t*_*s*_ switching from the stripe state to the gap state (blue symbols and line) increases linearly as the corresponding barrier height increases, but ln *t*_*s*_ switching from the gap state to the stripe state (red symbols) doesn't depend on the corresponding barrier height linearly, which cannot be explained solely by the barrier height. IntBH between potential basins in the gap state and the saddle point decreases very slowly as β increases (the red line in Figure 3A,B), suggesting that IntBH does not solely determine *t*_*s*_ in such situation. Apart from the barrier height, the distance (*d*_*w*_) between the closest potential basins also affects *t*_*s*_, particularly when the barrier height remains nearly unchanged. As shown in Figure 5F, it is found that all *d*_*w*_s increase as β increases. Consequently, the increase of *d*_*w*_ naturally leads to an increase of *t*_*s*_ from wells in the gap state to the ones in the stripe state. Furthermore, we plot *t*_*s*_ of both the inner‐state and inter‐state switching time in Figure 5E. We observe that as β increases, *t*_*s*_ mainly increases in the single phase of gap and stripe states and maintains a large value in the spot state, aligning with the behavior of InnBH as shown in Figure 3B. Therefore, the larger β leads to a more stable vegetation pattern, i.e., the ecosystem tends to maintain its configuration rather than change the orientation or phase shift. Additionally, the speed of spatial pattern switching not only depends on barrier height, but also on distance between basins.

### 2.3 Dynamical and Thermodynamical Mechanisms of the Critical Transition

Now we focus on the dynamical and thermodynamical mechanisms of the critical transition in the semi‐arid ecosystem, which undergoes a transition from one spatial pattern to another as β increases. To capture the dynamics of the ecosystem during the transition, we introduce the averaged flux *J*_*ave*_,^[^ ^31^, ^38^ ^]^ motivated by the way in which the nonequilibrium flux (decomposed from the driving force) can effectively characterize the nonequilibrium aspects of the dynamics. To understand the thermodynamics of the transition, we introduce the noise‐averaged global entropy production rate (EPR) *e*_*p*_.^[^ ^65^ ^]^ The EPR serves to quantify the degree of detailed balance breaking and time‐reversal symmetry in the mode space, or in other words, the extent to which the system is out of equilibrium. As the nonequilibrium steady state necessarily dissipates energy to sustain nonequilibrium conditions, the entropy flux directly correlates with the system's thermodynamic costs. Thus, EPR emerges as the key metric for characterizing the thermodynamics of spatially extended systems. (see Supporting Information for further details). *J*_*ave*_ and *e*_*p*_ can be written in terms of the steady‐state flux **J**_*ss*_ as presented in Equations (11) and (12) in the Experimental Section.

**Figure 6:**
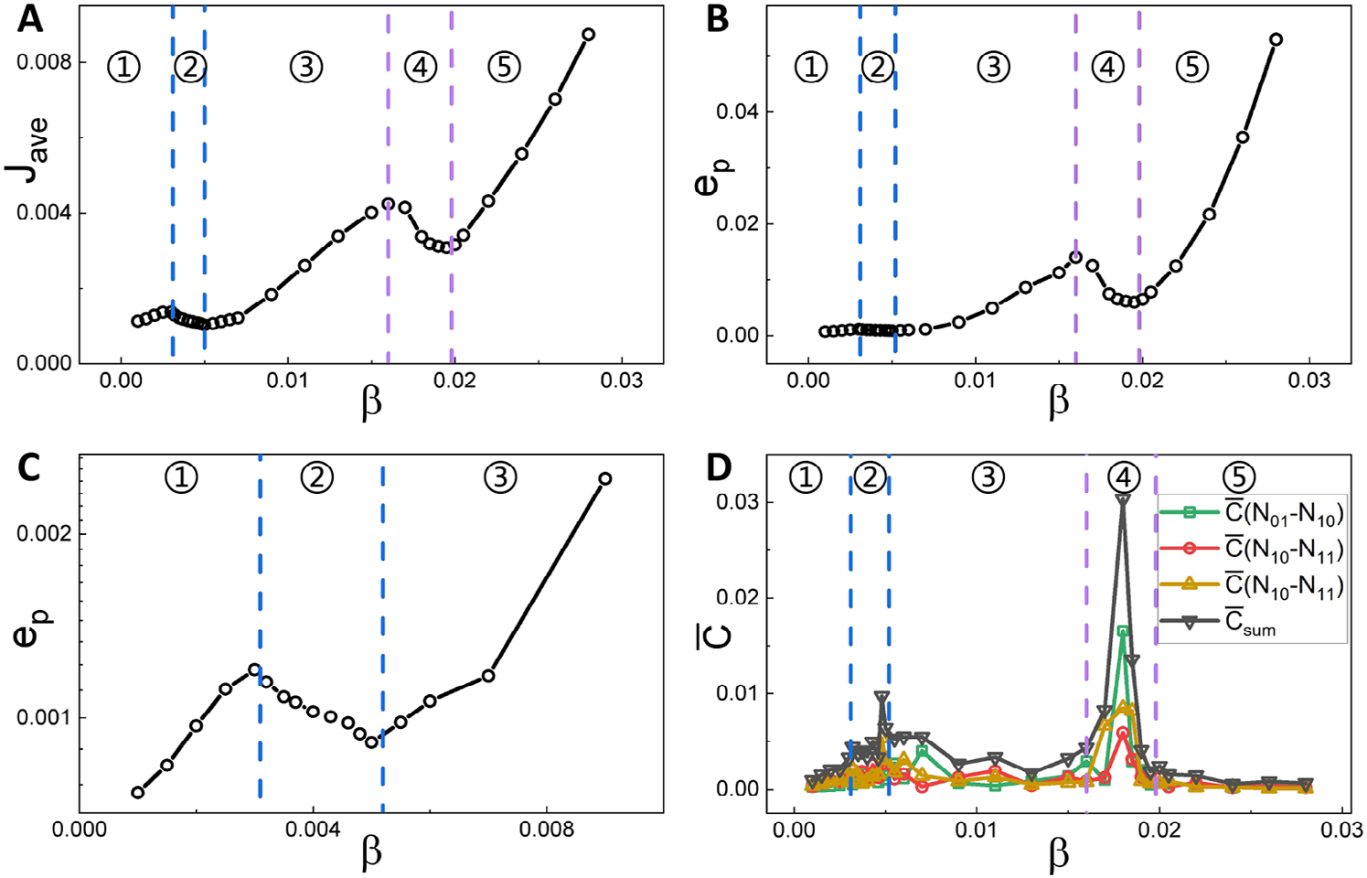
The dynamical and thermodynamical mechanisms of the critical transition in the semi‐arid ecosystem. A) Dependence of *J*_*ave*_ on β. B) Dependence of *e*_*p*_ on β. C) The zoomed‐in image of (B) at small β. (D) Dependence of $\bar{C}$ on β. The regions labeled with 1–5 represent the gap, gap/stripe, stripe, stripe/spot and spot states, respectively. The blue and purple dashed lines denote the phase boundaries.

**Figure** **6**A–C show *J*_*ave*_ and *e*_*p*_ as functions of β. We can observe that *J*_*ave*_ increases monotonically as β increases in the single phases but decreases in the coexisting phases, resulting in peaks appearing near the phase boundaries. The driving force for the nonequilibrium dynamics can be decomposed into the gradient force of the landscape and the curl force of the nonequilibrium flux.^[^ ^29^, ^31^ ^]^ The gradient force due to its convergent nature tends to attract the ecosystem down to the attractor and stabilize it. However, the flux, which breaks the detailed balance due to its rotational nature, is delocalized and favors global movements or switching instead of localizing in one point in the state space. Thus, the dynamical effect of the nonequilibrium flux is to lead to possible instability in one phase while stabilizing the continuous flow between phases. Consequently, the mechanism of the critical transition in the semi‐arid ecosystem can be attributed to the emergence of peaks in the averaged nonequilibrium flux. In other words, the nonequilibrium flux serves as the driving force or the dynamical origin for the critical transitions (or bifurcations) and thus the spatial pattern switching.

Similarly, *e*_*p*_ increases monotonically as β increases in the single phases and exhibits a general upward trend, indicating that as plant roots become more efficient in local water uptake, the ecosystem dissipates more, leading the system to be further away from equilibrium. Interestingly, *e*_*p*_ also exhibits peaks near the phase boundaries with maxima and minima appearing in the same qualitative pattern as for the averaged flux. Notably, switching from a single phase to a coexisting phase requires significant flux force to transform the one‐basin landscape to form the two‐basin landscapes, which results in a higher maintenance cost near the beginning boundaries of the critical transition or bifurcation. Thus, the entropy production we calculated in the particular mode space, representing the thermodynamic cost to sustain or vary the states, reflects the degree of detailed balance breaking and time reversal symmetry breaking of the ecosystem. Therefore, the emergence of peaks in nonequilibrium thermodynamic cost characterized by EPR provides an indicator for the thermodynamical mechanism of the critical transition in the semi‐arid ecosystem. In other words, the entropy production as nonequilibrium consumption/dissipation serves as the thermodynamic driving force or the thermodynamical origin for the critical transitions or bifurcations and thus the spatial pattern switching. Moreover, since spot vegetation patterns has been shown to be a potential signal of the onset of desertification in previous studies, both the averaged flux and EPR may serve as early warning signals for desertification.

Furthermore, we seek to understand whether the transitions between alternative stable states in this model are “smooth” or “abrupt” as parameters vary. Based on sections of the landscape (Figure 2F–J), we find that the potential landscape basins located in the spot state emerge suddenly at the beginning of the stripe/spot coexisting phase, whereas the ones located in the stripe state appear continuously at the beginning of the gap/stripe coexisting phase. Thus the critical transition from the stripe state to the spot state is discontinuous (the nucleation process), while the transition from the gap state to the stripe state is continuous or smooth. Additionally, both *J*_*ave*_ and *e*_*p*_ show a more pronounced decrease in the stripe/spot coexisting phase than in the gap/stripe coexisting phase. A sharper peak in EPR during a transition generally shows that this transition is more violent, leading to the same conclusions as those based on the landscape changes discussed above.

In addition, to measure the time irreversibility of the system in practice from time series observations, we introduce the average differences $\bar{C}$ between two‐point cross‐correlations forward *C*_1_ and backward *C*_2_ in time (see Supporting Information for definitions). As shown in Figure 6D, the obtained $\bar{C}$ of each pair of two modes and their summation show maximums in the coexisting phases, demonstrating that the time irreversibility of the system reaches its maximums during the transition process. And it is observed that $\bar{C}$ in the stripe/spot coexisting phase is much larger than the one in the gap/stripe coexisting phase, indicating that the time irreversibility in the former is larger than that in the later, further demonstrating that the transition from the stripe state to the spot state is more violent than the one from the gap state to the stripe state.

## 3 Discussion and Conclusion

In summary, we developed the landscape and flux field theory combined with the mode expansion method under appropriate truncations to the spatial vegetation patterns in semi‐arid ecosystems. The mode expansion method with appropriate truncations enabled us to reduce the substantial number of DOFs in spatial dynamics and identify several key spatial modes. As a consequence, the complex functional Fokker‐Planck equation for the probabilistic evolution for stochastic spatial dynamics can be transformed into a more tractable Fokker‐Planck equation in the mode space. Subsequently, by generalizing the landscape and flux theory to the spatial dynamics, not only the potential landscape of the ecosystem can be used to quantified the global stability via characterizing the states, their weights, and barrier height between states, but also the nonequilibrium flux field, as well as the EPR can be obtained, which serves to reveal the dynamical and thermodynamical mechanisms of semi‐arid ecosystems exhibiting different spatial patterns.

**Figure 7:**
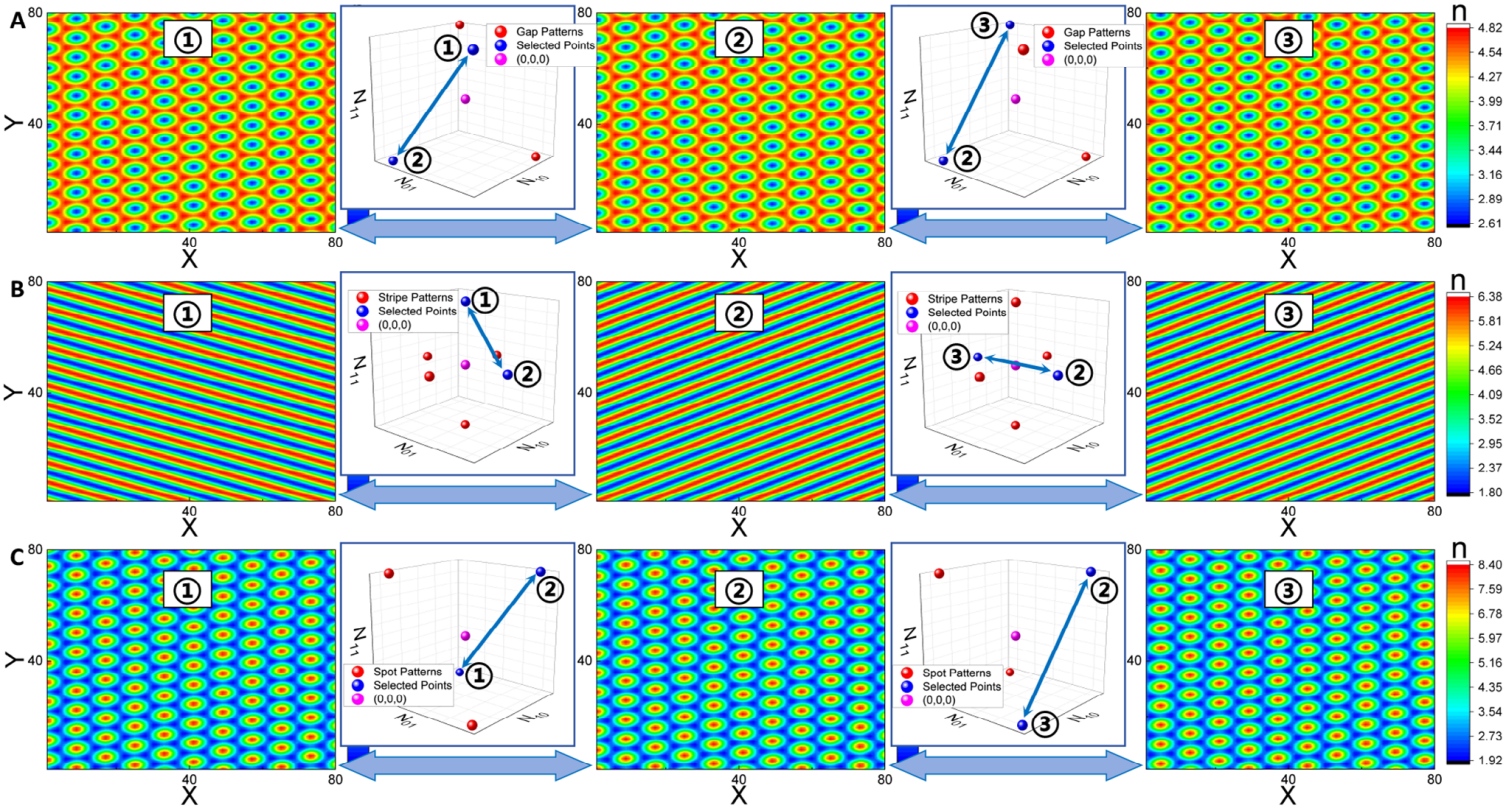
Schematic diagram that links mode amplitudes to spatial configurations. A–C) are respectively the gap, stripe and spot patterns, including three patterns with different orientations or phase shifts and two schematic diagrams exhibiting the corresponding modes' positions. The spatial pattern and its corresponding state in the mode space (the blue point) are marked with the same number.

Our findings show that as β increases, the landscape evolves successively from the potential basin located at gap to gap/stripe, stripe, stripe/spot, and finally spot spatial pattern states, consistent with the observed changes in vegetation patterns. These ecological spatial patterns connect directly with the amplitudes of spatial modes, To improve the accessibility and clarity of this connection, we plot a schematic diagram that links mode amplitudes to spatial configurations. As shown in **Figure** **7**, for both the gap and spot patterns (Figure 7A,C), when the system moves from one state to the other state (blue points in Figure 7A,C), the gap or spot pattern exhibits phase shifts of half a period along the directions defined by the vertices of the hexagonal lattice, but its shape or orientation won't change. For the stripe patterns (Figure 7B), when the system moves from one state to the other closest state (the two spatial configurations on the left), the stripe pattern changes its orientation. When the system moves from one state to the state at the opposite position (the two spatial configurations on the right), the stripe pattern only shows phase shifts but the same orientation.

**Figure 8:**
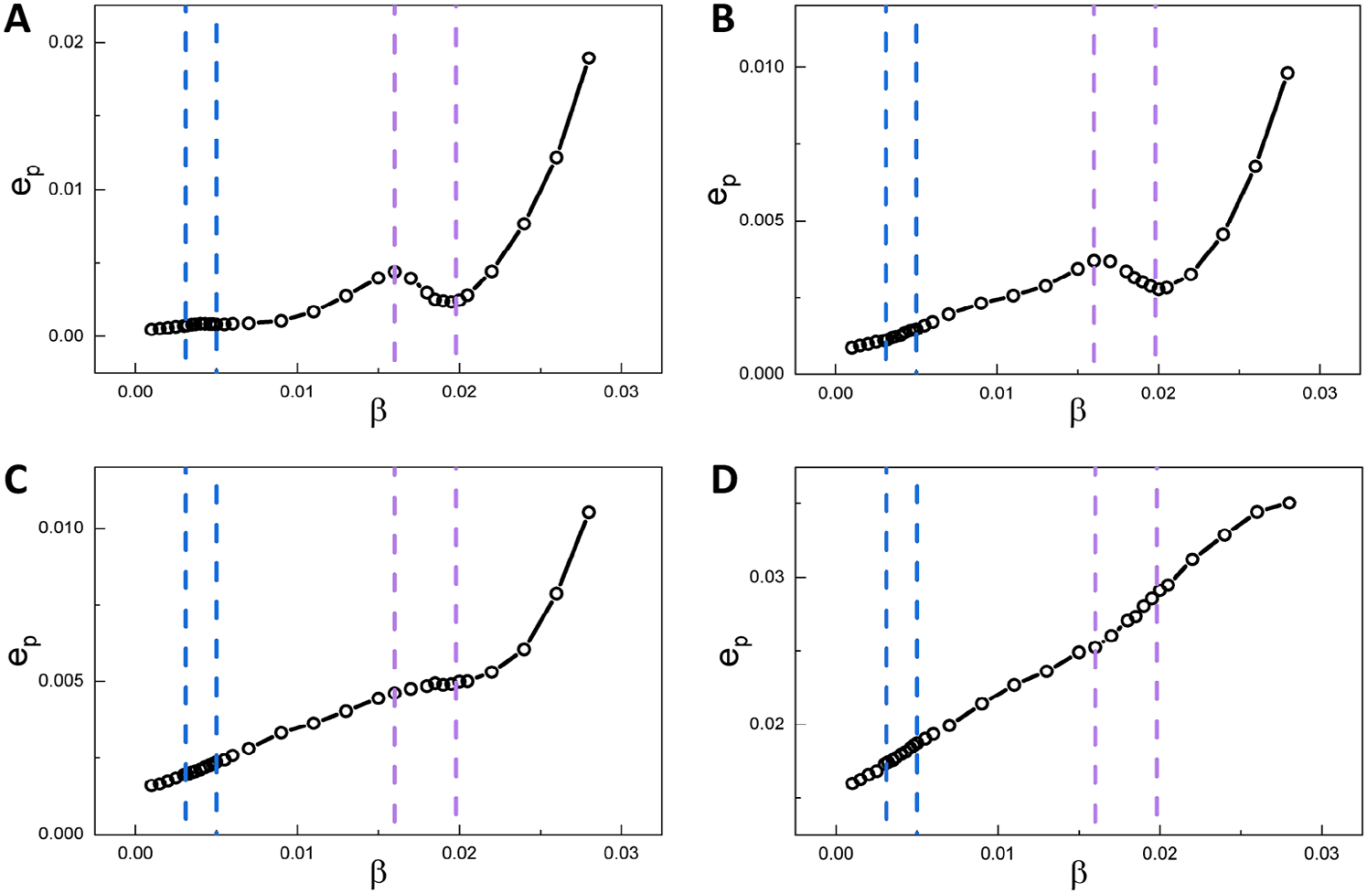
The influence of the ecosystem size (the noise intensity). Dependence of the entropy production rate *e*_*p*_ on β with (A) *k* = 17, *L*_*b*_ = 59.1, B) *k* = 13, *L*_*b*_ = 45.2, (C) *k* = 9, *L*_*b*_ = 31.3 and (D) *k* = 5, *L*_*b*_ = 17.4. The blue and purple dashed lines represent the phase boundaries between the single phases and the coexisting phases.

Moreover, our findings reveal that the nonequilibrium flux inside the landscape often points to the opposite direction of the potential gradient, acting as a driving force for the system to switch from one spatial pattern state to the another. The flux also contributes to the non‐overlapping nature of transition paths between potential basins located at different spatial pattern states, demonstrating the time‐reversal symmetry breaking of the ecosystem, further offering a new method to study the mechanism of pattern switching. Furthermore, both the averaged flux and EPR exhibit peaks near the phase boundaries, not only revealing the dynamical and thermodynamical mechanisms of the critical transition or bifurcation, but also serving as early warning signals for the desertification. Additionally, intensive simulations explored the influence of ecosystem size(noise), showing that as the size decreases(noise intensity increases), the critical transition shifts from a discontinuous to a continuous process, and may even disappear (**Figure** **8** and more details can be found in Supporting Information). The noise intensity which leads to the disappearance of the peak around the critical transition is the critical noise threshold. Beyond this threshold, the averaged flux or the EPR cannot sever as the early warning signals of spatial pattern switching. To further verify the generality and robustness of our method, we also varied other parameters and observed similar results (see Supporting Information and Figures S13 and S14, Supporting Information for details).

Compared to the existing methods such as the reaction‐diffusion equation (RDE) approach, the nonequilibrium landscape‐flux approach is more preferable for exploring spatial pattern (SP) foramtion and switching in nonequilibrium systems. Detials can be found in the following **Table** **1**.

**Table 1:** Comparison of the existing methods such as the reaction‐diffusion equation approach and the nonequilibrium landscape‐flux approach.

Research orientation	the RDE approach	the landscape‐flux approach
subject to stochastic forcing/noise	usually ignore	take into account
weight/probability of SP appearance	can not quantify	can quantify
the driving force for the dynamics	is given	decomposes as gradient+flux force
connect SPs	can not	can do so via barrier between basins
difficulty‐degree of switching SPs	can not give	can do so via barrier height
the speed estimate of SP switching	can not give	can do so via transitions between basins
the path taking for SP switching	can not estimate	can do so via path integral method
quantification of thermodynamic cost	doesn't address	provides it via EPR
physical mechanisms of SP switching	doesn't provide	provides it via change of flux/EPR
early warning signals for SP switching	doesn't provide	can do so via change of flux/EPR

While our current framework prioritizes the soil‐water diffusion feedback β as the principal control variable governing vegetation pattern dynamics, this methodology demonstrates inherent scalability for other ecological interactions which may affect the spatial pattern formation and switching. For example, when we try to study on the influence of the water input (rainfall), we only need to change the parameter *a* in Equation (1) and then apply the landscape‐flux theory with mode expansion method. However, if the ecological interactions (such as herbivory or nutrient cycling) can not be included in the semi‐arid model (Equation (1) in Experimental Section) in its current form, we need to find or set up a new model involving these ecological interactions. Such a new model would subsequently require re‐derivation of nonequilibrium potentials and flux fields through the mode expansion method. The influence of other ecological interactions on the spatial pattern formation and switching might be incorporated into future work.

Our findings are consistent with the real‐world vegetation patterns (such as aerial photographs presented in Figure S15, Supporting Information) in semi‐arid ecosystem, including many regions such as parts of Africa,^[^ ^49^, ^50^, ^51^, ^52^ ^]^ Australia,^[^ ^53^, ^54^ ^]^ and Mexico,^[^ ^55^ ^]^ as well as the observations that the vegetation states along the rainfall gradient or increasing β change from the hexagonal gap pattern to the stripe pattern, and finally to the hexagonal spot pattern.^[^ ^46^, ^48^, ^63^ ^]^ To translate our findings into actionable measures for ecosystem management, we first need to acquire the time evolution data of the high‐resolution spatial pattern from field observations in target real‐world semi‐arid regions, and then map these datasets onto the mode space in order to obtain the time trajectory of each mode. These trajectories can replace the simulation data performed based on the stochastic Langevin equations (Equation (7) in Experimental Section). Subsequently, we can construct nonequilibrium landscapes with flux fields of the system during different times and then calculate the averaged flux and the EPR for different times. Finally, we need to monitor the temporal evolution of these parameters. If the averaged flux or the EPR shows a sudden variation such as rising a peak, it provides an early warning signal for critical transitions of spatial pattern switching. This theoretical method and prediction may be validated through real‐world ecological data in future work.

The current study provides a convincing example to highlight the significant potential of utilizing the landscape and flux theory through the mode expansion method to nonequilibrium systems with spatial patterns formation and switching. Since spatial patterns exist widely in nature across physical, chemical, biological, ecological, and other fields, ranging from microscopic patterns in embryonic development to macroscopic patterns of vegetation or animal community, we believe that our work offers a significant and general approach to characterizing the pattern formation and switching in spatially extended systems, such as embryonic development or turbulence systems. This not only provides insights into understanding the driving forces, but also allows us to reveal the underlying mechanisms of formation and switching of spatial patterns, such as the critical transition or bifurcation from one pattern state to another one. In addition, our method may provide a useful tool for practical applications in real systems, for example, giving an early warning of desertification or forest fire.

## 4 Experimental Section

The focus was on the well‐known semi‐arid model of vegetation biomass and water^[^ ^46^ ^]^ with the soil‐water diffusion feedback^[^ ^47^, ^48^ ^]^ (see Supporting Information for details). By adding noise terms to the deterministic evolution Equation (S3) (Supporting Information), the stochastic partial differential equations (PDEs) of semi‐arid ecosystem model can be written as follows:

(1) $$\begin{array}{cc} \frac{\partial n}{\partial t} & =wn^{2}-mn+\Delta n+\zeta_{n}\left(t\right)\\ \frac{\partial w}{\partial t} & =a-w-wn^{2}+\alpha \Delta \left(w-\beta n\right)+\zeta_{w}\left(t\right) \end{array}$$

Herein, *w* and *n* represent the water and vegetation biomass, respectively; *a* controls water input; *m* measures plant losses; α represents the diffusion rate of water; and β denotes the water‐uptake ability of the roots, i.e., the soil‐water diffusion feedback intensity. ζ_*n*_ and ζ_*w*_ are stochastic terms with the time correlation satisfying with^[^ ^39^, ^66^ ^]^ $\left\langle \zeta \left(r,t\right)\zeta \left(r^{\prime },t^{\prime }\right)\right\rangle =\delta \left(t-t^{\prime }\right)\times M$, where ζ is a vector consisting of the components of ζ_*n*_ and ζ_*w*_, **M** is a 2 × 2 matrix:

(2) $$M=\left[\begin{array}{cc} \left(wn^{2}+mn\right)\delta \left(r-r^{\prime }\right) & -wn^{2}\delta \left(r-r^{\prime }\right)\\ +2\nabla \cdot \nabla^{\prime }\left[n\delta \left(r-r^{\prime }\right)\right] & \\ & \left(a+w+wn^{2}\right)\delta \left(r-r^{\prime }\right)\\ -wn^{2}\delta \left(r-r^{\prime }\right) & +2\alpha \nabla \cdot \nabla^{\prime }\left[\left(w-\beta n\right)\delta \left(r-r^{\prime }\right)\right] \end{array}\right]$$

*m* = 1.85, *a* = 8.0, α = 50.0 was fixed with reflecting boundary conditions. The parameters used in this study were derived and estimated from the published data in Ref. [48] (Details on the parameter selections can be found in Supporting Information). It was found that the vegetation pattern undergoes a progression from gap to stripe and ultimately spot shapes as β increases (see details in Figure S1, Supporting Information). These results demonstrated that β plays a very important role during the pattern formation and switching process in semi‐arid ecosystems.

Based on the stochastic PDEs (Equation (1)), the corresponding probability evolution is predictable and can be described as the functional Fokker‐Planck equation (FPE):

(3) $$\begin{array}{cc} \frac{\partial P}{\partial t} & =-\int_{0}^{L_{b}}dx\int_{0}^{L_{b}}dy\left\{\frac{\delta }{\delta n(x,y)}A_{n}+\frac{\delta }{\delta w(x,y)}A_{w}\right\}P+\\ & \frac{1}{2}\int_{0}^{L_{b}}dx\int_{0}^{L_{b}}dy\int_{0}^{L_{b}}dx^{\prime }\int_{0}^{L_{b}}dy^{\prime }\{\frac{\delta^{2}}{\delta n\left(x,y\right)\delta n\left(x^{\prime },y^{\prime }\right)}B_{nn}+\\ & \frac{\delta^{2}}{\delta w\left(x,y\right)\delta w\left(x^{\prime },y^{\prime }\right)}B_{ww}+2\frac{\delta^{2}}{\delta n\left(x,y\right)\delta w\left(x^{\prime },y^{\prime }\right)}B_{nw}\}P \end{array}$$

Herein, *A* and *B* are respectively the “convective” and “diffusion” parts, with specific expressions described as Equation (S6)(Supporting Information). However, exact solutions for Equation (3) remain almost impossible due to its huge numbers of DOFs in the spatial locations.

To address this, the mode expansion method^[^ ^43^, ^44^, ^45^ ^]^ was introduced to map these huge numbers of DOFs into spatial modes. Here, a Fourier transformation was performed and used the Fourier cosine transform to expand *n* and *w* in the spatial mode space: $n\left(x,y,t\right)=\sum_{i=0}^{+\infty }\sum_{j=0}^{+\infty }N_{ij}\left(t\right)\cos\left(\left(ix+jy\right)k\pi /L_{b}\right)$ and $w\left(x,y,t\right)=\sum_{i=0}^{+\infty }\sum_{j=0}^{+\infty }W_{ij}\left(t\right)\cos\left(\left(ix+jy\right)k\pi /L_{b}\right)$ (see details and explanations in Supporting Information). Hence, the functional FPE (Equation (3)) in the real space could be changed to the FPE in the spatial mode space based on the spatial modes (*N*_*ij*_, *W*_*ij*_) (see details and definitions of $\bar{A}$, $\bar{B}$ in Supporting Information):

(4) $$\begin{array}{cc} \frac{\partial P}{\partial t}= & -\sum_{i=0}^{+\infty }\sum_{j=0}^{+\infty }\left\{\frac{\partial {\bar{A}}_{n,ij}P}{\partial N_{ij}}+\frac{\partial {\bar{A}}_{w,ij}P}{\partial W_{ij}}\right\}+\frac{1}{2L_{b}^{2}}\sum_{i=0}^{+\infty }\sum_{j=0}^{+\infty }\sum_{i^{\prime }=0}^{+\infty }\sum_{j^{\prime }=0}^{+\infty }\\ & \left\{\frac{\partial^{2}{\bar{B}}_{nn,ij,i^{\prime }j^{\prime }}P}{\partial N_{ij}\partial N_{i^{\prime }j^{\prime }}}+2\frac{\partial^{2}{\bar{B}}_{nw,ij,i^{\prime }j^{\prime }}P}{\partial N_{ij}\partial W_{i^{\prime }j^{\prime }}}+\frac{\partial^{2}{\bar{B}}_{ww,ij,i^{\prime }j^{\prime }}P}{\partial W_{ij}\partial W_{i^{\prime }j^{\prime }}}\right\} \end{array}$$

Then appropriate truncations^[^ ^43^, ^44^, ^45^ ^]^ was set to select several key spatial modes, including *N*_00_, *N*_01_, *N*_10_, and *N*_11_ (components of **N**) as well as *W*_00_, *W*_01_, *W*_10_, and *W*_11_ (components of **W**) (see Supporting Information for details). The rationale of the truncation was to keep only the low lying or low energy modes and cut off all high energy excited modes, and it make sure that all kinds of Turing patterns including stripe or hexagonal even uniform shapes could be reproduced by these selected modes with truncations completely and easily. Considering solely the role of deterministic terms and ignoring the stochastic terms, the deterministic evolution of these truncated modes can be described as the following equations:

(5) $$\dot{N}=G\left(N,W\right),\dot{W}=H\left(N,W\right)$$

herein, **G** and **H** consist of functions $G_{N_{00}}$, $G_{N_{01}}$, $G_{N_{10}}$, $G_{N_{11}}$, and $H_{W_{00}}$, $H_{W_{01}}$, $H_{W_{10}}$, $H_{W_{11}}$ respectively, and their specific expressions are described as Equation (S10)–(S17) (Supporting Information). Simulations were performed based on these dynamic equations with *L*_*b*_ = 80 and *k* = 23 (see details in Supporting Information). As a result, it was found that as β increases, the vegetation pattern changes from uniform to gap, followed by stripe and finally spot pattern (see phase diagram and typical snapshots in Supporting Information and Figure S2, Supporting Information), consistent with the results obtained from simulations in the real space (Figure S1, Supporting Information).

Moreover, the time scales of relaxation for the diverse modes differ significantly. In Figure S3 (Supporting Information), it was evident that *N*_01_, *N*_10_ and *N*_11_ exhibit slower relaxation compared to the other five modes. Thus, after employing an adiabatic approximation, the other five modes could be expressed in terms of *N*_01_, *N*_10_, and *N*_11_, meaning that the dynamic equations only depend on these three slow‐changing modes. Therefore, Equation (4) can be simplified to the Fokker‐Planck equation based on *N*_01_, *N*_10_, and *N*_11_ (see details in Supporting Information):

(6) $$\frac{\partial P}{\partial t}=-\sum_{ij=01,10,11}\frac{\partial F_{N_{ij}}P}{\partial N_{ij}}+\frac{1}{2L_{b}^{2}}\sum_{ij=01,10,11}^{i^{\prime }j^{\prime }=01,10,11}\frac{\partial^{2}D_{ij,i^{\prime }j^{\prime }}P}{\partial N_{ij}\partial N_{i^{\prime }j^{\prime }}}$$

herein, $D_{ij,i^{\prime }j^{\prime }}$ is the element of the diffusion matrix with the expression described as Equations (S22)–(S27)(Supporting Information)_.

According to Equation (6), the stochastic Langevin equations in the mode space can read as (see parameters and details in Supporting Information):

(7) $$\begin{array}{cc} \frac{dN_{01}}{dt} & =F_{N_{01}}\left(N_{01},N_{10},N_{11}\right)+\xi_{N_{01}}\left(t\right)\\ \frac{dN_{10}}{dt} & =F_{N_{10}}\left(N_{01},N_{10},N_{11}\right)+\xi_{N_{10}}\left(t\right)\\ \frac{dN_{11}}{dt} & =F_{N_{11}}\left(N_{01},N_{10},N_{11}\right)+\xi_{N_{11}}\left(t\right) \end{array}$$

The landscape and flux theory^[^ ^28^, ^29^, ^30^, ^31^, ^32^, ^33^, ^34^, ^35^, ^36^, ^37^, ^38^ ^]^ can be applied in the mode space, and the effective potential *U* and the steady‐state nonequilibrium flux **J**_*ss*_ could be defined as (see details and parameter definitions in Supporting Information):

(8) $$U\left(N_{01},N_{10},N_{11}\right)=-\ln P_{ss}\left(N_{01},N_{10},N_{11}\right)$$

(9) $$J_{ss}=FP_{ss}-\nabla \cdot \left(DP_{ss}\right)$$

**Figure 9:**
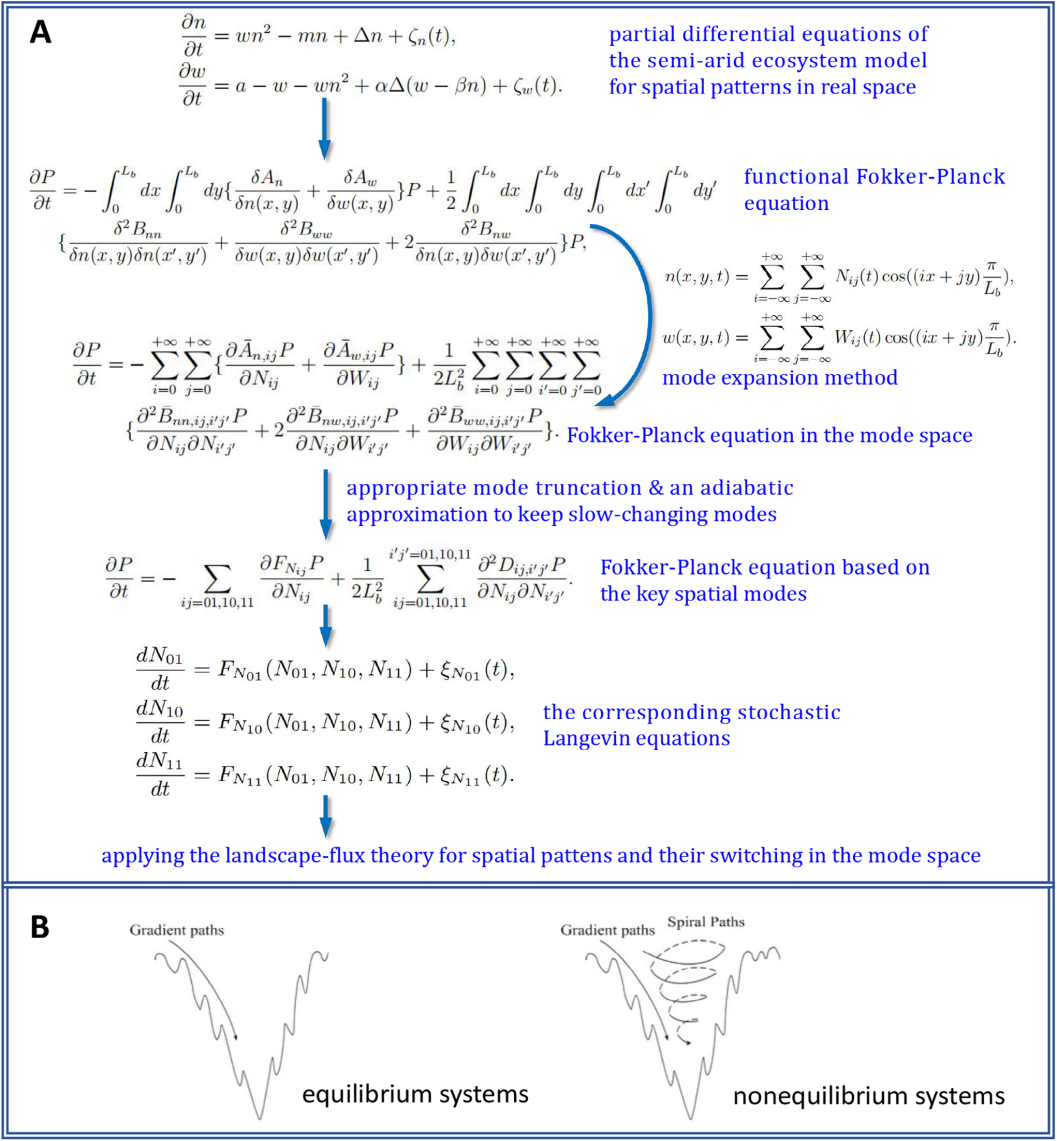
The landscape‐flux approach with mode expansion method applying to the semi‐arid ecosystem model. A) The flow chart of this theoretical framework and approach. B) Comparison of equilibrium dynamics with Boltzmann distribution landscape and nonequilibrium dynamics with gradient landscape&flux.

To improve the clarity of the approach, the flow chart in **Figure** **9**A is illustrated. Different from the case in equilibrium that the driving force was only related to the potential gradient (see the left picture of Figure 9B), i.e., the flux is equal to *zero*, In the steady state of the nonequilibrium system (see the right picture of Figure 9B), due to the non‐zero rotational flux field, the driving force (**F**) could be decomposed into the gradient part (**F**_*gradient*_), the curl part (**F**_*curl*_) and the part related to the spatial dependent noise (**F**_*D*_):

(10) $$F=F_{gradient}+F_{curl}+F_{D}=-D\cdot \nabla U+J_{ss}/P_{ss}+\nabla \cdot D$$

Furthermore, the averaged flux *J*_*ave*_ and the entropy production rate *e*_*p*_ are calculated to describe the dynamical and thermodynamical nature of the ecosystem (see details in Supporting Information):

(11) $$J_{ave}=\left\langle \right|J_{ss}\left(N_{01},N_{10},N_{11}\right)\left|\right\rangle$$

(12) $$e_{p}=\;\int \int \int \;dN_{01}dN_{10}dN_{11}\frac{J_{ss}^{T}\cdot D^{-1}\cdot J_{ss}}{P_{ss}}$$

## Conflict of Interest

The authors declare no conflict of interest.

## Author Contributions

J.W. and S.A.L. designed the research; J.S. and J.W. performed research; J.S., W.W., D.P., S.A.L., and J.W. contributed new analytic tools and analyzed data; All authors discussed the results and co‐wrote the manuscript.

## Supporting information

Supporting Information

## Data Availability

The data that support the findings of this study are available from the corresponding author upon reasonable request.

## References

[1] I. Lengyel , I. R. Epstein , Science 1991, 251, 650. 17741380 10.1126/science.251.4994.650

[2] I. Lengyel , I. R. Epstein , Proc. Natl. Acad. Sci. USA 1992, 89, 3977. 11607288 10.1073/pnas.89.9.3977 PMC525614

[3] J. E. Pearson , Science 1993, 261, 189. 17829274 10.1126/science.261.5118.189

[4] O. Pourquié , Science 2003, 301, 328. 12869750 10.1126/science.1085887

[5] X. Fan , Y. Fu , X. Zhou , L. Sun , M. Yang , M. Wang , R. Chen , Q. Wu , J. Yong , J. Dong , L. Wen , J. Qiao , X. Wang , F. Tang , Sci. Adv. 2020, 6, eaaz2978. 32923614 10.1126/sciadv.aaz2978 PMC7450478

[6] N. C. Lammers , V. Galstyan , A. Reimer , S. A. Medin , C. H. Wiggins , H. G. Garcia , Proc. Natl. Acad. Sci. USA 2020, 117, 836. 31882445 10.1073/pnas.1912500117 PMC6969519

[7] J. Briscoe , S. Small , Development 2015, 142, 3996. 26628090 10.1242/dev.129452 PMC4712844

[8] D. D. Patterson , S. A. Levin , C. Staver , J. D. Touboul , SIAM J. Appl. Dyn. Syst. 2020, 19, 2682.

[9] D. Patterson , S. Levin , A. C. Staver , J. Touboul , Bull. Math. Biol. 2024, 86, 3. 10.1007/s11538-023-01231-7 PMC10682166 38010440

[10] D. D. Patterson , A. C. Staver , S. A. Levin , J. D. Touboul , SIAM J. Appl. Math. 2023, S225. 10.1007/s11538-023-01231-7 PMC10682166 38010440

[11] G.‐Q. Sun , J. Zhang , L.‐P. Song , Z. Jin , B.‐L. Li , Appl. Math. Comput. 2012, 218, 11151.

[12] M. Banerjee , S. Abbas , Ecol. Complexity 2015, 21, 199.

[13] R. S. Cantrell , C. Cosner , Spatial ecology via reaction‐diffusion equations, John Wiley & Sons New York, New York 2004.

[14] M. Cross , H. Greenside , Pattern formation and dynamics in nonequilibrium systems, Cambridge University Press, Cambridge 2009.

[15] W. K. Smith , M. P. Dannenberg , D. Yan , S. Herrmann , M. L. Barnes , G. A. Barron‐Gafford , J. A. Biederman , S. Ferrenberg , A. M. Fox , A. Hudson , J. F. Knowles , N. MacBean , D. J. P. Moore , P. L. Nagler , S. C. Reed , W. A. Rutherford , R. L. Scott , X. Wang , J. Yang , Remote Sens. Environ. 2019, 233, 111401.

[16] Y. Zeng , D. Hao , A. Huete , B. Dechant , J. Berry , J. M. Chen , J. Joiner , C. Frankenberg , B. Bond‐Lamberty , Y. Ryu , J. Xiao , G. R. Asrar , M. Chen , Nat. Rev. Earth Environ. 2022, 3, 477.

[17] B. J. Chen , S. N. Teng , G. Zheng , L. Cui , S.‐p. Li , A. Staal , J. U. Eitel , T. W. Crowther , M. Berdugo , L. Mo , H. Ma , L. Bialic‐Murphy , C. M. Zohner , D. S. Maynard , C. Averill , J. Zhang , Q. He , J. B. Evers , N. P. R. Anten , H. Yizhaq , I. Stavi , E. Argaman , U. Basson , Z. Xu , M.‐J. Zhang , K. Niu , Q.‐X. Liu , C. Xu , J. Ecol. 2022, 110, 2268.

[18] C. E. Tarnita , J. A. Bonachela , E. Sheffer , J. A. Guyton , T. C. Coverdale , R. A. Long , R. M. Pringle , Nature 2017, 541, 398. 28102267 10.1038/nature20801

[19] O. Sizov , E. Ezhova , P. Tsymbarovich , A. Soromotin , N. Prihod'ko , T. Petäjä , S. Zilitinkevich , M. Kulmala , J. Bäck , K. Köster , Biogeosciences 2021, 18, 207.

[20] J. C. Rocha , M. M. Baraibar , L. Deutsch , A. de Bremond , J. S. Oestreicher , F. Rositano , C. C. Gelabert , Ecol. Soc. 2019, 24.

[21] Q. Ouyang , H. L. Swinney , Chaos: Interdiscipl. J. Nonlin. Sci. 1991, 1, 411. 10.1063/1.165851 12779937

[22] K. Gustavsson , B. Mehlig , Adv. Phys. 2016, 65, 1. 10.1140/epje/i2016-16055-0 27225619

[23] M. Rietkerk , R. Bastiaansen , S. Banerjee , J. van de Koppel , M. Baudena , A. Doelman , Science 2021, 374, eabj0359. 34618584 10.1126/science.abj0359

[24] X. Fu , Y. Zhao , J. I. Lopez , A. Rowan , L. Au , A. Fendler , S. Hazell , H. Xu , S. Horswell , S. T. C. Shepherd , C. E. Spencer , L. Spain , F. Byrne , G. Stamp , T. O'Brien , D. Nicol , M. Augustine , A. Chandra , S. Rudman , A. Toncheva , A. J. S. Furness , L. Pickering , S. Kumar , D.‐M. Koh , C. Messiou , D. ap Dafydd , M. R. Orton , S. J. Doran , J. Larkin , C. Swanton , et al., Nat. Ecol. Evol. 2022, 6, 88. 34949820 10.1038/s41559-021-01586-x PMC8752443

[25] V. Dakos , S. R. Carpenter , E. H. van Nes , M. Scheffer , Philos. Trans. R. Soc. Lond., B, Biol. Sci. 2015, 370, 20130263.

[26] S. Kéfi , V. Guttal , W. A. Brock , S. R. Carpenter , A. M. Ellison , V. N. Livina , D. A. Seekell , M. Scheffer , E. H. Van Nes , V. Dakos , PloS one 2014, 9, e92097. 24658137 10.1371/journal.pone.0092097 PMC3962379

[27] G. Zurlini , K. B. Jones , K. H. Riitters , B.‐L. Li , I. Petrosillo , Ecol. Indic. 2014, 45, 549.

[28] L. Xu , D. Patterson , S. A. Levin , J. Wang , Proc. Natl. Acad. Sci. USA 2023, 120, e2218663120. 36689655 10.1073/pnas.2218663120 PMC9945981

[29] J. Wang , L. Xu , E. Wang , Proc. Natl. Acad. Sci. U. S. A. 2008, 105, 12271. 18719111 10.1073/pnas.0800579105 PMC2527901

[30] C. Li , J. Wang , Proc. Natl. Acad. Sci. U. S. A. 2014, 111, 14130. 25228772 10.1073/pnas.1408628111 PMC4191801

[31] X. Fang , K. Kruse , T. Lu , J. Wang , Rev. Mod. Phys. 2019, 91, 045004.

[32] X. Chu , J. Wang , Appl. Phys. Rev. 2020, 7, 031403. 32884608 10.1063/5.0007316 PMC7376616

[33] J. Wang , Adv. Phys. 2015, 64, 1.

[34] J. Su , Z. Cao , J. Wang , H. Jiang , Z. Hou , Cell Rep. Phys. Sci. 2024, 5, 101817.

[35] X. Fang , J. Wang , Annu. Rev. Biophys. 2020, 49, 227. 32375020 10.1146/annurev-biophys-121219-081656

[36] C. Li , J. Wang , Cancer Res. 2015, 75, 2607. 25972342 10.1158/0008-5472.CAN-15-0079

[37] H. Yan , L. Zhao , L. Hu , X. Wang , E. Wang , J. Wang , Proc. Natl. Acad. Sci. USA 2013, 110, E4185. 24145451 10.1073/pnas.1310692110 PMC3831465

[38] L. Xu , D. Patterson , A. C. Staver , S. A. Levin , J. Wang , Proc. Natl. Acad. Sci. USA 2021, 118, e2103779118. 34117123 10.1073/pnas.2103779118 PMC8214705

[39] W. Wu , J. Wang , J. Phys. Chem. B 2013, 117, 12908. 23865936 10.1021/jp402064y

[40] W. Wu , J. Wang , J. Chem. Phys. 2013, 139. 10.1063/1.4816376 24089732

[41] W. Wu , J. Wang , J. Chem. Phys. 2014, 141. 10.1063/1.4894389 25217956

[42] W. Wu , J. Wang , Front. Phys. 2020, 8, 567523.

[43] M. E. Peskin , An introduction to quantum field theory, CRC press, Boca Raton 2018.

[44] C. Itzykson , J.‐M. Drouffe , Statistical field theory: volume 2, strong coupling, Monte Carlo methods, conformal field theory and random systems, vol. 2, Cambridge University Press, Cambridge 1991.

[45] L. Wettmann , M. Bonny , K. Kruse , Interface Focus 2014, 4, 20140039. 25485085 10.1098/rsfs.2014.0039 PMC4213450

[46] C. A. Klausmeier , Science 1999, 284, 1826. 10364553 10.1126/science.284.5421.1826

[47] J. von Hardenberg , E. Meron , M. Shachak , Y. Zarmi , Phys. Rev. Lett. 2001, 87, 198101. 11690457 10.1103/PhysRevLett.87.198101

[48] G.‐Q. Sun , C.‐H. Wang , L.‐L. Chang , Y.‐P. Wu , L. Li , Z. Jin , Appl. Math. Modell. 2018, 61, 200.

[49] C. Valentin , J.‐M. d'Herbès , J. Poesen , Catena 1999, 37, 1

[50] G. Worrall , J. Soil Sci. 1959, 10, 34.

[51] L. P. White , J. Ecol. 1970, 549.

[52] A. J. Belsky , Vegetatio 1987, 70, 51.

[53] J. A. Mabbutt , P. C. Fanning , J. Arid. Environ. 1987, 12, 41.

[54] J. Ludwig , D. Tongway , K. Hodgkinson , D. Freudenberger , J. Noble , Landscape ecology, function and management: principles from Australia's rangelands, Csiro Publishing, Australia 1996.

[55] C. Montana , J. Lopez‐Portillo , A. Mauchamp , J. Ecol. 1990, 789.

[56] M. Rietkerk , S. C. Dekker , P. C. De Ruiter , J. van de Koppel , Science 2004, 305, 1926. 15448261 10.1126/science.1101867

[57] E. Sheffer , J. von Hardenberg , H. Yizhaq , M. Shachak , E. Meron , Ecol. Lett. 2013, 16, 127. 23157578 10.1111/ele.12027

[58] M. Scheffer , S. Carpenter , J. A. Foley , C. Folke , B. Walker , Nature 2001, 413, 591. 11595939 10.1038/35098000

[59] S. Kéfi , M. Rietkerk , C. L. Alados , Y. Pueyo , V. P. Papanastasis , A. ElAich , P. C. De Ruiter , Nature 2007, 449, 213. 17851524 10.1038/nature06111

[60] M. Scheffer , J. Bascompte , W. A. Brock , V. Brovkin , S. R. Carpenter , V. Dakos , H. Held , E. H. Van Nes , M. Rietkerk , G. Sugihara , Nature 2009, 461, 53. 19727193 10.1038/nature08227

[61] E. Meron , E. Gilad , J. Von Hardenberg , M. Shachak , Y. Zarmi , Chaos, Solitons & Fractals 2004, 19, 367.

[62] E. Meron , Math. Modell. Nat. Phenom. 2011, 6, 163.

[63] E. Meron , Nonlinear physics of ecosystems, CRC Press, Taylor & Francis Group Boca Raton, FL, USA 2015.

[64] J. Wang , K. Zhang , E. Wang , J. Chem. Phys. 2010, 133. 10.1063/1.3478547 20886967

[65] U. Seifert , Rep. Prog. Phys. 2012, 75, 126001. 23168354 10.1088/0034-4885/75/12/126001

[66] C. W. Gardiner et al., Handbook of stochastic methods, Vol. 3, Springer Berlin, Berlin 1985.

