# Supplementary material for: Revealing Physical Mechanisms of Spatial Pattern Formation and Switching in Ecosystems via Nonequilibrium Landscape and Flux
Source: Adv Sci (Weinh). 2025 Apr 25;12(26):2501776. doi: 10.1002/advs.202501776 (PMC12245119; doi:10.1002/advs.202501776)
Supplement: Supplementary file 1 — Supporting Information [file ADVS-12-2501776-s001.pdf]

ADVANCED SCIENCE
Open Access

Supporting Information

for *Adv. Sci.*, DOI 10.1002/advs.202501776

Revealing Physical Mechanisms of Spatial Pattern Formation and Switching in Ecosystems via Nonequilibrium Landscape and Flux

Jie Su, Wei Wu, Denis D. Patterson, Simon Asher Levin and Jin Wang**

Supplementary Information: Revealing Physical Mechanisms of Spatial Pattern Formation and Switching in Ecosystems via Nonequilibrium Landscape and Flux

Jie Su[1], Wei Wu[1], Denis Patterson[2,3,4], Simon Asher Levin[2,3],* and Jin Wang[1,5†]

1.Center for Theoretical Interdisciplinary Sciences, Wenzhou Institute,

University of Chinese Academy of Sciences, Wenzhou 325001, China

2.High Meadows Environmental Institute,

Princeton University, Princeton, NJ 08544, USA

3.Department of Ecology and Evolutionary Biology,

Princeton University, Princeton, NJ 08544, USA

4.Department of Mathematical Sciences,

Durham University, Durham, UK and

5.Department of Chemistry and of Physics and Astronomy,

State University of New York of Stony Brook,

Stony Brook, New York 11794, USA

(Dated: March 12, 2025)

SUPPLEMENTARY TEXT

The semi-arid model of vegetation biomass and water

The following model of vegetation biomass and water located in a two-dimensional semi-arid ecosystem with a finite size L_b was first proposed by Klausmeier [1]:

$$\begin{aligned}\frac{\partial W}{\partial T} &= A - LW - RWN^2 + V\frac{\partial W}{\partial X},\\ \frac{\partial N}{\partial T} &= JRWN^2 - MN + D(\frac{\partial^2}{\partial X^2} + \frac{\partial^2}{\partial Y^2})N.\end{aligned} \tag{S1}$$

Herein, W and N represent the water and vegetation biomass, respectively. In the first equation, A denotes the uniform supply rate of the water, LW is the evaporation rate and RWN^2 represents the rate that plants take up water. The last term denotes the surface run-off, indicating that water flows downhill at speed V in the negative direction of the X axis. In the second equation, J is the the energy transfer coefficient, meaning the yield of the vegetation biomass per unit water consumed. MN denotes the density-independent natural mortality of plants. The last term is the diffusion rate of plants with D the diffusion coefficient, representing the rate of plant movement by clonal growth or seed dispersal. We rescale the system as follows: $w = \sqrt{R/L}JW$, $n = \sqrt{R/L}N$, $a = \sqrt{R/L^3}JA$, $t = LT$, $m = M/L$, $v = V/\sqrt{DL}$, $x = \sqrt{L/D}X$, and $y = \sqrt{L/D}Y$. This model then can be normalized to the following form [1]:

$$\begin{aligned}\frac{\partial w}{\partial t} &= a - w - wn^2 + v\frac{\partial w}{\partial x},\\ \frac{\partial n}{\partial t} &= wn^2 - mn + \Delta n,\end{aligned} \tag{S2}$$

where $\Delta = \frac{\partial^2}{\partial x^2} + \frac{\partial^2}{\partial y^2}$ is the general Laplace operator.

Taking the soil-water diffusion feedback into consideration, von Hardenberg et al. [2] added a cross-diffusion term $\alpha\Delta(w - \beta n)$ into the equation of water in Eq.S2. This cross-diffusion term describes the water transport influenced by the plant's roots taking up water locally, and the parameter β represents the water-uptake ability of the roots. In other words, the large β will lead to a large soil-water gradient generated between the plant and the surrounding patch. Such effect can be called soil-water diffusion feedback and β characterizes this feedback intensity. Because the slope of hill is not the essential condition

* Corresponding Author: slevin@princeton.edu

† Corresponding Author: jin.wang.1@stonybrook.edu

for the emergence of vegetation patterns [3], we set $v = 0$ so that the semi-arid model of vegetation biomass and water with the soil-water diffusion feedback can be described as follows [3]:

$$\begin{aligned} \frac{\partial n}{\partial t} &= wn^2 - mn + \Delta n, \\ \frac{\partial w}{\partial t} &= a - w - wn^2 + \alpha \Delta (w - \beta n). \end{aligned} \tag{S3}$$

We fix $m = 1.85$, $a = 8.0$, $\alpha = 50.0$ (details on the parameter selections can be found in the next section), and start simulations from random initial configurations and with reflecting boundary conditions. As a result, spatial vegetation patterns dependent on different β can be obtained based on Eq.S3. We perform simulations of the ecosystem with $L_b = 80$ and the obtained vegetation patterns in the steady state are shown in Fig.S1. For small β such as $\beta = 0.001$ (Fig.S1A), the vegetation pattern appears uniform. As β slightly increases to 0.003 (Fig.S1B), the pattern changes to be gap shape. With further increases in β to 0.005 (Fig.S1C) and continuing 0.017 (Fig.S1D), the pattern transforms into stripe shape. Finally, for larger β such as 0.018 (Fig.S1E) and 0.024 (Fig.S1F), the vegetation pattern evolves into spot shape. To summarize, the vegetation undergoes a progression from uniform to gap, then stripe and ultimately spot patterns as β increases.

Details on the model parameter selections

The nondimensionalized model (Eq.S2) has three parameters: a, which controls water input; m, which measures plant losses; and v, which controls the rate at which water flows downhill. Klausmeier provided the plausible values of these parameter as follows: $a_{tree} =$ 0.077 to 0.23, $m_{tree} = 0.045$, $a_{grass} = 0.94$ to 2.81, $m_{grass} = 0.45$, and $v = 182.5$ [1]. In this study, the soil-water diffusion feedback is taken into consideration, so the cross-diffusion term is added into the equation of water. Moreover, because the slope of hill is not the essential condition for the emergence of vegetation patterns [3], v is set to 0. Therefore, the nondimensionalized model (Eq.S3) has four parameters: a, which controls water input; m, which measures plant losses; α, which represents the diffusion rate of water; and β, which denotes the water-uptake ability of the roots. Due to the ecosystem changes from hills to plains, the value ranges of a and m will have a shift. According to the bifurcation diagram (Fig.1, a ranges from 0 to 10) and three sets of model parameters (Table 2, 1.$a = 3.5$, $m = 1.3$, $\alpha = 50$; 2.$a = 4.4$, $m = 1.8$, $\alpha = 10$; 3.$a = 5.5$, $m = 1.3$, $\alpha = 50$) in Ref.[3],

we assumed that the plausible values of the model parameters need to satisfy the following conditions: $a < 10$, $m < 2a$. Finally, three sets of model parameters are selected as follows: 1.$a = 8$, $m = 1.85$, $\alpha = 50$; 2.$a = 4.4$, $m = 1.8$, $\alpha = 10$; 3.$a = 4.8$, $m = 1.95$, $\alpha = 10$.

The Functional Fokker-Planck equation from the stochhastic model

To obtain the functional Fokker-Planck equation of the semi-arid ecosystem in the spatial location, we first need to add the stochastic terms to Eq.S3:

$$\begin{aligned} \frac{\partial n}{\partial t} &= wn^2 - mn + \Delta n + \zeta_n(t), \\ \frac{\partial w}{\partial t} &= a - w - wn^2 + \alpha\Delta(w - \beta n) + \zeta_w(t). \end{aligned} \tag{S4}$$

where ζ_n and ζ_w are stochastic terms with the time correlation satisfying with [4, 5] $\langle \boldsymbol{\zeta}(\mathbf{r}, t)\boldsymbol{\zeta}(\mathbf{r}', t')\rangle = \delta(t - t')\times$

$$\begin{bmatrix} (wn^2 + mn)\delta(\mathbf{r} - \mathbf{r}') + 2\nabla \cdot \nabla'[n\delta(\mathbf{r} - \mathbf{r}')] & -wn^2\delta(\mathbf{r} - \mathbf{r}') \\ -wn^2\delta(\mathbf{r} - \mathbf{r}') & (a + w + wn^2)\delta(\mathbf{r} - \mathbf{r}') + 2\alpha\nabla \cdot \nabla'[(w - \beta n)\delta(\mathbf{r} - \mathbf{r}')] \end{bmatrix}$$

where $\boldsymbol{\zeta}$ is a vector consisting of the components of ζ_n and ζ_w. Hence, the resulting functional Fokker-Planck equation for the probability functional evolution $P[n(x, y), w(x, y), t]$ can be written as follows:

$$\begin{aligned} \frac{\partial P}{\partial t} = &- \int_0^{L_b} dx \int_0^{L_b} dy \{\frac{\delta}{\delta n(x, y)} A_n + \frac{\delta}{\delta w(x, y)} A_w\}P + \frac{1}{2}\int_0^{L_b} dx \int_0^{L_b} dy \int_0^{L_b} dx' \int_0^{L_b} dy' \\ &\{\frac{\delta^2}{\delta n(x, y)\delta n(x', y')} B_{nn} + \frac{\delta^2}{\delta w(x, y)\delta w(x', y')} B_{ww} + 2\frac{\delta^2}{\delta n(x, y)\delta w(x', y')} B_{nw}\}P \end{aligned} \tag{S5}$$

with

$$\begin{aligned} A_n &= \dot{n} = wn^2 - mn + \nabla^2 n, \\ A_w &= \dot{w} = a - w - wn^2 + \alpha\nabla^2(w - \beta n), \\ B_{nn} &= (wn^2 + mn)\delta(\mathbf{r} - \mathbf{r}') + 2\nabla \cdot \nabla'[n\delta(\mathbf{r} - \mathbf{r}')], \\ B_{ww} &= (a + w + wn^2)\delta(\mathbf{r} - \mathbf{r}') + 2\alpha\nabla \cdot \nabla'[(w - \beta n)\delta(\mathbf{r} - \mathbf{r}')], \\ B_{nw} &= -wn^2\delta(\mathbf{r} - \mathbf{r}'). \end{aligned} \tag{S6}$$

Mode expansion method applied on the semi-arid ecosystem

In order to reduce the large number of DOFs in spatial locations, we employ the mode expansion method [6–8] to map these DOFs into several key spatial modes. For a linear stability analysis of the homogeneous stationary solution, we can expand the vegetation biomass n and water w in the orthogonal spatial plane coordinate system as follows:

$$\begin{aligned} n(x,y,t) &= \sum_{i=-\infty}^{+\infty}\sum_{j=-\infty}^{+\infty} N_{ij}(t)\cos((ix+jy)\frac{\pi}{L_b}), \\ w(x,y,t) &= \sum_{i=-\infty}^{+\infty}\sum_{j=-\infty}^{+\infty} W_{ij}(t)\cos((ix+jy)\frac{\pi}{L_b}). \end{aligned} \tag{S7}$$

However, both the gap and spot patterns (Fig.S1B,E,F) are located at the vertices of the hexagonal lattice. Therefore, it is more appropriate to apply the mode expansion method in the hexagonal plane coordinate system:

$$\begin{aligned} n(x',y',t) &= \sum_{i=-\infty}^{+\infty}\sum_{j=-\infty}^{+\infty} N_{ij}(t)\cos\left((ix'+jy')\frac{\pi}{L_b}\right), \\ w(x',y',t) &= \sum_{i=-\infty}^{+\infty}\sum_{j=-\infty}^{+\infty} W_{ij}(t)\cos\left((ix'+jy')\frac{\pi}{L_b}\right) \end{aligned} \tag{S8}$$

with $x' = \frac{\sqrt{3}}{2}x - \frac{1}{2}y$ and $y' = y$. Herein, x or y has (has not) the superscript representing that the coordinate system is the spatial hexagonal (orthogonal) plane.

We now make a Galerkin ansatz and truncate the expansion series formula Eq.S8 at a finite value. The rationale is to keep only the low lying or low energy modes and cut off all high energy excited modes. Since the vegetation patterns belong to the Turing patterns with striped and hexagonal structures, it is sufficient to truncate at $|\cos([\frac{\sqrt{3}}{2}i + (j - \frac{1}{2}i)]| \leq 1$, i.e., the truncated modes consist of N_{00}, N_{01}, N_{10}, N_{11}, N_{0-1}, N_{-10}, N_{-1-1} and the same W as in the N subscript. All types of spatial Turing patterns including stripe or hexagonal even uniform shape can be reproduced and represented by these modes easily (as shown in Fig.S2). These spatial patterns are highly consistent with those generated from evolution equations in the real space (Fig.S1), demonstrating that the truncation is suitable, and these selected spatial modes can capture the principal pattern characteristics of the spatial patterns in the real space. Although using additional modes might improve the degree of accuracy, it

is not expected to qualitatively alter the pattern dynamics. And using additional modes would introduce unnecessary complexity for our current theoretical framework. Practical applications may benefit from additional modes for more quantitative accuracies. But this is beyond the scope of the current study. Moreover, due to the symmetry of hexagonal coordinates, the mode with subscripts $-i$ and $-j$ can be merged into the mode with subscript i and j, so modes with subscript including negative value can be ignored. Additionally, it is observed that the spatial patterns generated by the evolution equation (Eq.S3) exhibit periodicity (Fig.S1). However, the truncated model based on $\cos((ix' + jy')\pi/L_b)$ generates a pattern that covers only half a period. To address this limitation, we introduce a positive integer parameter k to rescale the pattern, ensuring alignment with the periodicity observed in spatial patterns generated by Eq.S3. By adjusting the base from $\cos((ix' + jy')\pi/L_b)$ to $\cos((ix' + jy')k\pi/L_b)$, the revised model will produce patterns with $k/2$ periods. Therefore, the expansion with the truncation in Eq.S8 reads as follows:

$$\begin{aligned} n(x', y', t) =& N_{00}(t) + B_{01}(t)\cos(\frac{k\pi y'}{L_b}) + N_{10}(t)\cos(\frac{k\pi x'}{L_b}) + N_{11}(t)\cos(\frac{k\pi(x'+y')}{L_b}),\\ w(x', y', t) =& W_{00}(t) + W_{01}(t)\cos(\frac{k\pi y'}{L_b}) + W_{10}(t)\cos(\frac{k\pi x'}{L_b}) + W_{11}(t)\cos(\frac{k\pi(x'+y')}{L_b}). \end{aligned} \tag{S9}$$

Inserting these expressions Eq.S9 into Eq.S3 and integrating with respect to x' and y', we obtain the following dynamic mode equations:

$$\begin{aligned} \dot{N}_{00} =& W_{00}N_{00}^2 + W_{01}N_{00}N_{01} + W_{00}N_{01}^2/2 + W_{10}N_{00}N_{10} + W_{11}N_{01}N_{10}/2 + W_{00}N_{10}^2/2 + \\ & W_{11}N_{00}N_{11} + W_{10}N_{01}N_{11}/2 + W_{01}N_{10}N_{11}/2 + W_{00}N_{11}^2/2 - mN_{00}, \end{aligned} \tag{S10}$$

$$\begin{aligned} \dot{N}_{01} =& -(\frac{k\pi}{L_b})^2 N_{01} + W_{01}N_{00}^2 + 2W_{00}N_{00}N_{01} + 3/4W_{01}N_{01}^2 + W_{11}N_{00}N_{10} + W_{10}N_{01}N_{10} + \\ & 1/2W_{01}N_{10}^2 + W_{10}N_{00}N_{11} + W_{11}N_{01}N_{11} + W_{00}N_{10}N_{11} + 1/2W_{01}N_{11}^2 - mN_{01}, \end{aligned} \tag{S11}$$

$$\begin{aligned} \dot{N}_{10} =& -(\frac{k\pi}{L_b})^2 N_{10} + W_{10}N_{00}^2 + W_{11}N_{00}N_{01} + 1/2W_{10}N_{01}^2 + 2W_{00}N_{00}N_{10} + W_{01}N_{01}N_{10} + \\ & 3/4W_{10}N_{10}^2 + W_{01}N_{00}N_{11} + W_{00}N_{01}N_{11} + W_{11}N_{10}N_{11} + 1/2W_{10}N_{11}^2 - mN_{10}, \end{aligned} \tag{S12}$$

$$\dot{N}_{11} = -(\frac{k\pi}{L_b})^2 N_{11} + W_{11}N_{00}^2 + W_{10}N_{00}N_{01} + 1/2W_{11}N_{01}^2 + W_{01}N_{00}N_{10} + W_{00}N_{01}N_{10} + 1/2W_{11}N_{10}^2 + 2W_{00}N_{00}N_{11} + W_{01}N_{01}N_{11} + W_{10}N_{10}N_{11} + 3/4W_{11}N_{11}^2 - mN_{11}, \tag{S13}$$

$$\dot{W}_{00} = a - W_{00} - (W_{00}N_{00}^2 + W_{01}N_{00}N_{01} + W_{00}N_{01}^2/2 + W_{10}N_{00}N_{10} + W_{11}N_{01}N_{10}/2 + W_{00}N_{10}^2/2 + W_{11}N_{00}N_{11} + W_{10}N_{01}N_{11}/2 + W_{01}N_{10}N_{11}/2 + W_{00}N_{11}^2/2), \tag{S14}$$

$$\dot{W}_{01} = -\alpha(\frac{k\pi}{L_b})^2(W_{01} - \beta N_{01}) - W_{01} - (W_{01}N_{00}^2 + 2W_{00}N_{00}N_{01} + 3/4W_{01}N_{01}^2 + W_{11}N_{00}N_{10} + W_{10}N_{01}N_{10} + 1/2W_{01}N_{10}^2 + W_{10}N_{00}N_{11} + W_{11}N_{01}N_{11} + W_{00}N_{10}N_{11} + 1/2W_{01}N_{11}^2), \tag{S15}$$

$$\dot{W}_{10} = -\alpha(\frac{k\pi}{L_b})^2(W_{10} - \beta N_{10}) - W_{10} - (W_{10}N_{00}^2 + W_{11}N_{00}N_{01} + 1/2W_{10}N_{01}^2 + 2W_{00}N_{00}N_{10} + W_{01}N_{01}N_{10} + 3/4W_{10}N_{10}^2 + W_{01}N_{00}N_{11} + W_{00}N_{01}N_{11} + W_{11}N_{10}N_{11} + 1/2W_{10}N_{11}^2), \tag{S16}$$

$$\dot{W}_{11} = -\alpha(\frac{k\pi}{L_b})^2(W_{11} - \beta N_{11}) - W_{11} - (W_{11}N_{00}^2 + W_{10}N_{00}N_{01} + 1/2W_{11}N_{01}^2 + W_{01}N_{00}N_{10} + W_{00}N_{01}N_{10} + 1/2W_{11}N_{10}^2 + 2W_{00}N_{00}N_{11} + W_{01}N_{01}N_{11} + W_{10}N_{10}N_{11} + 3/4W_{11}N_{11}^2). \tag{S17}$$

Phase diagram and typical snapshots of simulations in the mode space

We then perform simulations based on the dynamic equations Eq.S10-S17 with $L_b = 80$ and $k = 23$, started from 10000 different initial states where N_{00} and W_{00} are random numbers belonging to $[0, 5]$ and other modes are in the range $[0, 3]$. Utilizing the final states of modes in Eq.S9, the spatial patterns can be obtained.

As a result, the dependence of vegetation pattern probabilities P on β is illustrated in Fig.S2A. As β increases, the probabilities of the gap (H_π anti-hexagonal lattice state), stripe (B state) and spot (H_0 hexagonal lattice state) pattern approach 1 successively, indicating

the sequential appearance of these three patterns with the increase of β, proved by the typical snapshot of vegetation changing from uniform (Fig.S2B) to gap (Fig.S2C), followed by stripe (Fig.S2D) and finally spot pattern (Fig.S2E). It is noticed that the gap and spot patterns share the identical topological structure, but the difference lies in the distribution of minimum (gap) or maximum (spot) values on the vertices of a hexagonal lattice, i.e., the gap pattern forms anti hexagonal lattice and the spot pattern forms hexagonal lattice. All these results are consistent with the ones obtained from simulations in the real space (Fig.S1). In addition, it is found that between single phases of gap/stripe (stripe/spot) patterns, there exists a coexisting phase comprising these two patterns, implying that different initial states would lead to different spatial patterns.

Fokker-Planck equation in the mode space by applying the mode expansion method

To obtain the Fokker-Planck equation in the mode space, we first need to express the functional derivatives with respect to $n(x,y)$ and $w(x,y)$ in terms of partial derivatives with respect to N_{ij} and W_{ij}. Considering of $\delta n(x,y) = \delta N_{ij}\cos((ix+jy)\pi/L_b)$, if $\bar{F}$ represents the function of N_{ij} and W_{ij} corresponding to a functional F[n(x,y),c(x,y)], then we have:

$$
\begin{aligned}
\frac{\partial \bar{F}}{\partial N_{ij}} &= \int_0^{L_b} dx \int_0^{L_b} dy \frac{\delta F}{\delta n(x,y)} \cos((ix+jy)\pi/L_b),\\
\frac{\partial \bar{F}}{\partial W_{ij}} &= \int_0^{L_b} dx \int_0^{L_b} dy \frac{\delta F}{\delta w(x,y)} \cos((ix+jy)\pi/L_b),
\end{aligned}
\tag{S18}
$$

therefore,

$$
\begin{aligned}
\frac{\delta F}{\delta n(x,y)} &= \frac{2}{L_b^2}\sum_{i,j=0}^{\infty}\frac{\partial \bar{F}}{\partial N_{ij}}\cos((ix+jy)\frac{\pi}{L_b}),\\
\frac{\delta F}{\delta w(x,y)} &= \frac{2}{L_b^2}\sum_{i,j=0}^{\infty}\frac{\partial \bar{F}}{\partial W_{ij}}\cos((ix+jy)\frac{\pi}{L_b}),\\
\frac{\delta^2 F}{\delta n(x,y)\delta n(x',y')} &= \frac{4}{L_b^4}\sum_{i,j,i',j'=0}^{\infty}\frac{\partial^2 \bar{F}}{\partial N_{ij}\partial N_{i'j'}}\cos((ix+jy)\frac{\pi}{L_b})\cos((i'x'+j'y')\frac{\pi}{L_b}),\\
\frac{\delta^2 F}{\delta w(x,y)\delta w(x',y')} &= \frac{4}{L_b^4}\sum_{i,j,i',j'=0}^{\infty}\frac{\partial^2 \bar{F}}{\partial W_{ij}\partial W_{i'j'}}\cos((ix+jy)\frac{\pi}{L_b})\cos((i'x'+j'y')\frac{\pi}{L_b}),\\
\frac{\delta^2 F}{\delta n(x,y)\delta w(x',y')} &= \frac{4}{L_b^4}\sum_{i,j,i',j'=0}^{\infty}\frac{\partial^2 \bar{F}}{\partial N_{ij}\partial W_{i'j'}}\cos((ix+jy)\frac{\pi}{L_b})\cos((i'x'+j'y')\frac{\pi}{L_b}),
\end{aligned}
\tag{S19}
$$

We take Eq.S19 into the functional Fokker-Planck equation Eq.S5 and replace the base $\cos(\pi/L_b)$ into $\cos(k\pi/L_b)$. Then integrating with respect to x, y, x' and y', the functional Fokker-Planck equation in the real spcae can be changed to the Fokker-Planck equation in the mode space:

$$
\begin{aligned}
\frac{\partial P}{\partial t} = &-\sum_{i=0}^{+\infty}\sum_{j=0}^{+\infty}\{\frac{\partial \bar{A}_{n,ij}P}{\partial N_{ij}}+\frac{\partial \bar{A}_{w,ij}P}{\partial W_{ij}}\}+\frac{1}{2L_b^2}\sum_{i=0}^{+\infty}\sum_{j=0}^{+\infty}\sum_{i'=0}^{+\infty}\sum_{j'=0}^{+\infty}\\
&\{\frac{\partial^2 \bar{B}_{nn,ij,i'j'}P}{\partial N_{ij}\partial N_{i'j'}}+2\frac{\partial^2 \bar{B}_{nw,ij,i'j'}P}{\partial N_{ij}\partial W_{i'j'}}+\frac{\partial^2 \bar{B}_{ww,ij,i'j'}P}{\partial W_{ij}\partial W_{i'j'}}\}.
\end{aligned}
\tag{S20}
$$

$\bar{A}$ and $\bar{B}$ are respectively the "convective" and "diffusion" parts. By inserting Eq.S9 into the expressions of A_n and A_w in Eq.S6 and then integrating with respect to x' and y', we can obtain $\bar{A}_{n,ij} = \dot{N}_{ij}$ and $\bar{A}_{w,ij} = \dot{W}_{ij}$ with some specific expression as shown in Eq.S10-S17. Similarly, by inserting Eq.S9 into the expressions of B_{nn}, B_{nw} and B_{ww} in Eq.S6 and then integrating with respect to x' and y', we can obtain the expressions of $\bar{B}_{nn,ij,i'j'}$, $\bar{B}_{nw,ij,i'j'}$ and $\bar{B}_{ww,ij,i'j'}$ (we will give specific expressions in the next section).

The Fokker-Planck equation and the stochastic Langevin equations based on the key spatial modes

Except for the appropriate truncations applied in the mode expansion method (Eq.S9), we also find that the time scales of relaxation for the diverse modes differ significantly. In

Fig.S3, it is evident that N_{01}, N_{10} and N_{11} exhibit slower relaxation compared to the other five modes. Thus, after employing an adiabatic approximation, the other five modes can be expressed in terms of N_{01}, N_{10} and N_{11}, meaning that the dynamic equations only depend on these three slow-changing modes. Therefore, Eq.S20 can be transformed to a simpler Fokker-Planck equation only based on N_{01}, N_{10} and N_{11}:

$$\frac{\partial P}{\partial t} = -\sum_{ij=01,10,11} \frac{\partial F_{N_{ij}} P}{\partial N_{ij}} + \frac{1}{2L_b^2} \sum_{ij=01,10,11}^{i'j'=01,10,11} \frac{\partial^2 D_{ij,i'j'} P}{\partial N_{ij} \partial N_{i'j'}}. \tag{S21}$$

Hence, we arrive at Fokker-Planck equation based on the 3 slow-changing modes. Herein, $F_{N_{01}}$, $F_{N_{10}}$ and $F_{N_{11}}$ are the deterministic driving force which are respectively equal to $\dot{N}_{01}$, $\dot{N}_{10}$ and $\dot{N}_{11}$ with expressions in terms of Eq.S11-S13. And 9 elements of the diffusion matrix $D_{ij,i'j'}$ ($\equiv \bar{B}_{nn,ij,i'j'}$) can be expressed as follows:

$$D_{01,01} = 2W_{00}N_{00}^2 + 3W_{01}N_{00}N_{01} + 3/2W_{00}N_{01}^2 + 2W_{10}N_{00}N_{10} + 3/2W_{11}N_{01}N_{10} + W_{00}N_{10}^2 + 2W_{11}N_{00}N_{11} + 3/2W_{10}N_{01}N_{11} + 3/2W_{01}N_{10}N_{11} + W_{00}N_{11}^2 + 2mN_{00}, \tag{S22}$$

$$D_{10,10} = 2W_{00}N_{00}^2 + 2W_{01}N_{00}N_{01} + W_{00}N_{01}^2 + 3W_{10}N_{00}N_{10} + 3/2W_{11}N_{01}N_{10} + 3/2W_{00}N_{10}^2 + 2W_{11}N_{00}N_{11} + 3/2W_{10}N_{01}N_{11} + 3/2W_{01}N_{10}N_{11} + W_{00}N_{11}^2 + 2mN_{00}, \tag{S23}$$

$$D_{11,11} = 2W_{00}N_{00}^2 + 2W_{01}N_{00}N_{01} + W_{00}N_{01}^2 + 2W_{10}N_{00}N_{10} + 3/2W_{11}N_{01}N_{10} + W_{00}N_{10}^2 + 3W_{11}N_{00}N_{11} + 3/2W_{10}N_{01}N_{11} + 3/2W_{01}N_{10}N_{11} + 3/2W_{00}N_{11}^2 + 2mN_{00}, \tag{S24}$$

$$D_{01,10} = D_{10,01} = -2(\frac{k\pi}{L_b})^2 N_{11} + W_{11}N_{00}^2 + 2W_{10}N_{00}N_{01} + 3/4W_{11}N_{01}^2 + 2W_{01}N_{00}N_{10} + 2W_{00}N_{01}N_{10} + 3/4W_{11}N_{10}^2 + 2W_{00}N_{00}N_{11} + 3/2W_{01}N_{01}N_{11} + 3/2W_{10}N_{10}N_{11} + 3/4W_{11}N_{11}^2 + mN_{11} \tag{S25}$$

$$D_{01,11} = D_{11,01} = -2(\frac{k\pi}{L_b})^2 N_{10} + W_{10}N_{00}^2 + 2W_{11}N_{00}N_{01} + 3/4W_{10}N_{01}^2 + 2W_{00}N_{00}N_{10} + 3/2W_{01}N_{01}N_{10} + 3/4W_{10}N_{10}^2 + 2W_{01}N_{00}N_{11} + 2W_{00}N_{01}N_{11} + 3/2W_{11}N_{10}N_{11} + 3/4W_{10}N_{11}^2 + mN_{10}, \tag{S26}$$

$$D_{10,11} = D_{11,10} = -2(\frac{k\pi}{L_b})^2 N_{01} + W_{01}N_{00}^2 + 2W_{00}N_{00}N_{01} + 3/4W_{01}N_{01}^2 + 2W_{11}N_{00}N_{10} +$$
$$3/2W_{10}N_{01}N_{10} + 3/4W_{01}N_{10}^2 + 2W_{10}N_{00}N_{11} + 3/2W_{11}N_{01}N_{11} + 2W_{00}N_{10}N_{11} + 3/4W_{01}N_{11}^2 + mN_{01} \tag{S27}$$

On this basis, the stochastic Langevin equation of each mode then can be written as follows:

$$\begin{aligned}
\frac{dN_{01}}{dt} &= F_{N_{01}}(N_{01}, N_{10}, N_{11}) + \xi_{N_{01}}(t), \\
\frac{dN_{10}}{dt} &= F_{N_{10}}(N_{01}, N_{10}, N_{11}) + \xi_{N_{10}}(t), \\
\frac{dN_{11}}{dt} &= F_{N_{11}}(N_{01}, N_{10}, N_{11}) + \xi_{N_{11}}(t).
\end{aligned} \tag{S28}$$

The functions $F_{N_{01}}$, $F_{N_{10}}$ and $F_{N_{11}}$ are the components of deterministic driving force $\mathbf{F}$, which are respectively equal to $\dot{N}_{01}$, $\dot{N}_{10}$ and $\dot{N}_{11}$ with expressions in terms of Eq.S11-S13. Since the other five modes are now expressed in terms of N_{01}, N_{10} and N_{11}, we need to first fix N_{01}, N_{10}, N_{11} and calculate the other five modes by applying Eq.S10,S14-S17 until they remain nearly unchanged in simulations. Finally, $\xi_{N_{01}}$, $\xi_{N_{10}}$ and $\xi_{N_{11}}$ are the stochastic forces with time correlations satisfying $\langle \boldsymbol{\xi}(t)\boldsymbol{\xi}(t')\rangle = 2\mathbf{D}\delta(t-t')$, where $\boldsymbol{\xi}$ is a vector consisting of the components of $\xi_{N_{01}}$, $\xi_{N_{10}}$ and $\xi_{N_{11}}$. $\mathbf{D}$ is a 3×3 matrix representing the diffusion matrix, consisting of the intensity $1/2L_b^2$ and 9 elements $D_{ij,i'j'}$ with the expressions described as Eq.S22-S27.

Based on the stochastic equations of motion Eq.S28, we perform numerical simulations in the mode space with fixed parameters $m = 1.85$, $a = 8.0$, $\alpha = 50.0$, $L_b = 80$ and $k = 23$, if not otherwise stated. 10^6 simulations are run from different random initial configurations for a long enough time ($t_1 = 500$) with time step $\Delta t = 10^{-3}$ to ensure that each system reaches the steady state, and then they are run for another long time ($t_2 = 500$) for data sampling and analysis.

Details of the landscape and flux theory

The landscape and flux theory, which is widely applied in the nonequilibrium systems such as cell cycle, dynamics of neural networks, cancer, ecological systems, etc [9–20].

The evolution of a physical quantity $\mathbf{x}$ can be written as:

$$\dot{\mathbf{x}} = \mathbf{F}(\mathbf{x}) + \boldsymbol{\xi}(t). \tag{S29}$$

Herein, $\mathbf{F}$ is the deterministic driving force while $\boldsymbol{\xi}$ is the stochastic force with $\langle \boldsymbol{\xi}(t) \rangle = 0$ and having the time correlation which is satisfying with $\langle \boldsymbol{\xi}(t)\boldsymbol{\xi}(t') \rangle = 2\mathbf{D}\delta(t-t')$ with $\mathbf{D}$ the diffusion coefficient tensor. When the equilibrium systems reaches the steady state, the probability distribution $P(\mathbf{x})$ will remain constant, i.e., $\partial P_{ss}(\mathbf{x},t)/\partial t = 0$ (the subscript ss denotes the steady state). The effective potential related to the steady-state probability can be naturally defined as $U(\mathbf{x}) = -\ln P_{ss}(\mathbf{x})$. Since $\partial P_{ss}(\mathbf{x},t)/\partial t = 0$, it is found that the steady-state flux $\mathbf{J}_{ss}$ must be equal to 0 via the Fokker-Planck equation. Therefore, in the equilibrium system, the driving force in Eq.S29 only depends on the gradient of the effective potential, i.e., $\mathbf{F}(\mathbf{x}) = -\mathbf{D} \cdot \nabla U(\mathbf{x})$.

However, when the system is nonequilibrium, $\nabla \cdot \mathbf{J}(\mathbf{x},t) = 0$ does not mean that $\mathbf{J}$ has to vanish. In general, the flux $\mathbf{J}$ can exist in the form of a rotational curl or more precisely recurrent field, which can be described as follows in the steady state [10]:

$$\mathbf{J}_{ss}(\mathbf{x}) = \mathbf{F}(\mathbf{x})P_{ss}(\mathbf{x}) - \nabla_{\mathbf{x}} \cdot [\mathbf{D}P_{ss}(\mathbf{x})]. \tag{S30}$$

Similarly, the effective nonequilibrium potential can also be defined on the basis of the steady-state probability distribution P_{ss} like the one in the equilibrium system, so we have:

$$U(\mathbf{x}) = -\ln P_{ss}(\mathbf{x}). \tag{S31}$$

Since the steady-state flux doesn't vanish, it will contribute to the driving force so that the driving force $\mathbf{F}$ in Eq.S29 does not depend solely on the gradient of the nonequilibrium potential U_{neq} any more. $\mathbf{F}$ can be decomposed into the gradient part ($\mathbf{F}_{gradient}$), the curl part ($\mathbf{F}_{curl}$) and the part related to the spatial dependent noise ($\mathbf{F}_D$):

$$\mathbf{F} = \mathbf{F}_{gradient} + \mathbf{F}_{curl} + \mathbf{F}_D = -\mathbf{D} \cdot \nabla U + \mathbf{J}_{ss}/P_{ss} + \nabla \cdot \mathbf{D}. \tag{S32}$$

Hence, by applying the landscape and flux theory, we can not only understand the global information of the system by establishing the potential landscape, but also investigating dynamical and thermodynamical natures via calculating the flux, force contribution and entropy production rate, etc.

Detailed description of the degenerate steady states of each spatial pattern and their relationships with the positions (amplitudes) of key modes

By analysing the deterministic dynamics of N_{01}, N_{10} and N_{11} (i.e., Eq.S11-S13), the steady states of the semi-arid ecosystems with different β can be obtained. As shown in Fig.S4, final steady states (red spheres) along with 1000 randomly selected initial states (blue spheres) for various β corresponding to typical patterns are obtained, which are in agreement with the results from dynamic equations of all modes (Fig.S2).

It is noted that different locations in the mode space (i.e., different amplitudes of modes) represent distinct spatial patterns. For example, when $\beta = 0.001$ (Fig.S4A), the final state is located at the origin of the mode space (i.e., $N_{01} = N_{10} = N_{11} = 0$), which represents the uniform pattern (Fig.S1A and Fig.S2B). We will discuss the degenerate steady states of gap, stripe and spot patterns and their relationships with the positions (amplitudes) of key modes in the following part.

For the gap pattern such as $\beta = 0.003$ (Fig.S4B), there exist 4 steady states (H_π state) situated at the vertices of a regular tetrahedron with vertices having equal proportions to $(-1, -1, -1)$, $(-1, 1, 1)$, $(1, -1, 1)$ and $(1, 1, -1)$, respectively. In other words, when (N_{01}, N_{10}, N_{11}) is equal to $(-q, -q, -q)$, $(-q, q, q)$, $(q, -q, q)$ or $(q, q, -q)$ (q is a positive number), and according to Eq.S9, it is found that the spatial pattern is gap shape. These steady states are degenerate with equal probability or potential on the landscape, and patterns located in them all have a gap shape but exhibit phase shifts between them. As shown in the left-bottom picture of Fig.1 in the main text and Fig.S5, it is observed that compared to the selected pattern, the remaining 3 patterns exhibit phase shifts of half a period along the directions defined by the vertices of the hexagonal lattice. To improve the accessibility and clarity of the model connection to actual ecological patterns, we plot a schematic diagram that links mode amplitudes to spatial configurations. As shown in Fig.S8A, when the system moves from one state to the other state (blue points in Fig.S8A), the gap pattern exhibits a phase shift but its shape or orientation won't change.

For the stripe pattern such as $\beta = 0.009$ (Fig.S4D), it can be observed that 6 degenerate steady states (B state) with equal probability are situated at the regular octahedron vertices on the axis. In other words, when (N_{01}, N_{10}, N_{11}) is equal to $(0, 0, q)$, $(0, 0, -q)$, $(0, q, 0)$, $(0, -q, 0)$, $(q, 0, 0)$ or $(-q, 0, 0)$ (q is a positive number), and according to Eq.S9, it is found that the spatial pattern is stripe shape. As depicted in the middle-bottom picture of Fig.1

in the main text and Fig.S6, stripe patterns show diverse orientations when the states are located in different axes and present same orientation but different phase shifts when the states are located in the same axis. Specifically, when the system moves from one state to the other closest state (the two spatial configurations on the left in Fig.S8B), the stripe pattern changes its orientation. When the system moves from one state to the state at the opposite position (the two spatial configurations on the right in Fig.S8B), the stripe pattern only shows a phase shift but keeps the same orientation.

For the spot pattern such as $\beta = 0.026$ (Fig.S4F), the locations of 4 steady states (H_0 state) are the same as the ones of the gap pattern except for the vertices having equal proportions to $(1, 1, 1)$, $(-1, -1, 1)$, $(-1, 1, -1)$ and $(1, -1, -1)$, respectively. In other words, when (N_{01}, N_{10}, N_{11}) is equal to (q, q, q), $(-q, -q, q)$, $(-q, q, -q)$ or $(q, -q, -q)$ (q is a positive number), and according to Eq.S9, it is found that the spatial pattern is spot shape. Similarly, patterns located in these 4 degenerate states with equal probability all have a spot shape but exhibit phase shifts between them (the right-bottom picture of Fig.1 in the main text and Fig.S7). Similar to the case of the gap pattern, when the system moves from one state to the other state (blue points in Fig.S8C), the spot pattern exhibits a phase shift but its shape or orientation won't change.

Also, there exists two coexisting phases consisting of the gap/stripe patterns and the stripe/spot patterns, with typical examples presented in Fig.S4C and E at $\beta = 0.0043$ and $\beta = 0.018$, respectively. It is found that the steady states of the coexisting phases in Fig.S3C and E are the superpositions of H_π and B states for the former, H_0 and B states for the latter.

Entropy production

In this work, the evolution happened in the mode phase space rather than the positional space, so we need to calculate the entropy production of the mode's evolution in such phase space, based on the conventional stochastic thermodynamics framework [21]. The coupled dynamics of each mode evolves according to the following overdamped Langevin equation:

$$\dot{\mathbf{x}} = \mathbf{F}(\mathbf{x}) + \boldsymbol{\xi}(t), \tag{S33}$$

where $\mathbf{x} = (N_{01}, N_{10}, N_{11})^{\mathrm{T}}$, and $\mathbf{F}(\mathbf{x})$ is the deterministic driving force. $\boldsymbol{\xi}$ is the Gaussian white noise with the average value $\langle \boldsymbol{\xi}(t) \rangle = 0$ and the time correlation satisfying with

$\langle \boldsymbol{\xi}(\mathbf{x}, t)\boldsymbol{\xi}(\mathbf{x}, t')\rangle = 2\mathbf{D}\delta(t-t')$, where $\mathbf{D}$ is the diffusion tensor. On this basis, the probability distribution $P(\mathbf{x}, t)$ of the system in the mode space will evolve through the following Fokker-Planck equation:

$$\boldsymbol{\partial_t P(\mathbf{x}, t) = -\nabla \cdot \mathbf{J}(\mathbf{x}, t)}, \tag{S34}$$

where the probability current $\mathbf{J}(\mathbf{x}, t) = \mathbf{F}(\mathbf{x})P(\mathbf{x}, t) - \nabla \cdot (\mathbf{D}P(\mathbf{x}, t))$.

The trajectory-dependent stochastic entropy of the ecosystem reads as $s_{sys}(t) = -\ln P(\mathbf{x}, t)$, with average

$$S_{sys}(t) = -\int d\mathbf{x}P(\mathbf{x}, t)\ln P(\mathbf{x}, t). \tag{S35}$$

The change rate of the entropy S_{sys} can be decomposed into two contributions as $\dot{S}_{sys} = e_p - \dot{S}_e$. Herein, $\dot{S}_e$ is the change rate of the entropy flow rate from the medium to the system and e_p is the total entropy production rate (EPR) [21]. The explicit expression of EPR can be described as follows:

$$e_p = \int d\mathbf{x}\mathbf{J}^{\mathrm{T}}\mathbf{D}^{-1}\mathbf{J}/P, \tag{S36}$$

and the entropy flow rate from the medium to the system is $\dot{S}_e = \int d\mathbf{x}\mathbf{J}^{\mathrm{T}}\mathbf{D}^{\mathbf{-1}}\left(\mathbf{F} - \nabla \cdot \mathbf{D}\right)$ [14]. In the steady state of nonequilibrium systems, the temporal derivative of S_{sys} vanishes ($\dot{S}_{sys} \to 0$), rendering e_p (related to nonequilibrium driving force for the dynamics characterized by the flux) equivalent to $\dot{S}_e$ (related to work and dissipation).

There is another formula for the EPR: $e_p = -dF/dt + Q_{hk}$ [22]. The first term is the (time dependent) free energy relaxation and the second term is the “housekeeping heat”. At the steady state, the total EPR is equal to Q_{hk}, which denotes the dissipation heat and energy to maintain the system in the steady state.

It is known that the nonequilibrium open system will dissipate energy and cause entropy in the steady state because it has to exchange energy and information with the surroundings. Therefore, the EPR is closely related to the energy dissipation of the system, which can be used to characterize the thermodynamics of the nonequilibrium system.

Dominant modes in the nucleation process of the critical transition

We focus on the nucleation behavior in the discontinuous transition exemplified by $\beta = 0.018$ located in the B/H_0 coexisting phase. Since the transition is from the single phase of B state to that of the H_0 state, the nucleation process is the emergence of spot patterns located in the H_0 state. Taking the nucleation process for $\beta = 0.018$ as an example, the ecosystem switches from the B state with the potential well located in the region $N_{01} = N_{10} = 0$, $N_{11} > 0$ to the H_0 state with the well located in the region $N_{01} = N_{10} = N_{11} > 0$ (depicted by the black line in Fig.4C in the main text). We illustrate all modes at the beginning point (B state), the ending point (H_0 state) and the saddle point of the transition path in Fig.S10A. It is observed that N_{01} (red bars) and N_{10} (blue bars) shift from 0 to 0.94 and finally to 1.44, while N_{11} (green bars) changes from 2.28 to 1.98 and ultimately to 1.44, undergoing more obvious changes than the other modes, indicating that the dominant modes during the nucleation process are within these three modes. We then calculate the relative distance $\Delta = |N^s - N^e|/|N^b - N^e|$ for these three modes, where N includes N_{01}, N_{10} and N_{11} with superscripts b, s, e representing the beginning point, the saddle point and the ending point, respectively. Δ for N_{01}, N_{10} or N_{11} describes the distance of each mode between the saddle point and the ending point, normalized by the distance between the beginning and ending points. Notably, if Δ for the given mode is close to 1 ($N^s \approx N^b$), this mode at the saddle point become proximate to the beginning state, thus it is irrelevant. Conversely, if Δ for the given mode is close to 0 ($N^s \approx N^e$), this mode at the saddle point is close to the ending point, indicating its crucial role as nucleation seed, which can be readily identified. As illustrated in Fig.S10B, it is found that the relative distances of N_{01} and N_{10} (red and blue bars) are much shorter than the one of N_{11} (green bar), representing that N_{01} and N_{10} play dominant roles in the nucleation process on this transition path.

To elucidate the influence of the dominant modes N_{01} and N_{10} on the nucleation process, we present snapshots of vegetation patterns during the process based on the mode space (Fig.S11A-F). These vegetation patterns are represented by the vegetation biomass n from Eq.S9 with varying spatial modes on the transition path. As the ecosystem switches from the B state for the stripe pattern to the transition state at the saddle point, the vegetation biomass magnitude in regions of the hexagonal lattice increases rapidly, while the pattern still maintains its stripe shape (Fig.S11B-D). As the ecosystem further evolves beyond the saddle point ($N_{01} = N_{10} = 0.94$, $N_{11} = 1.98$, Fig.S11D) along the transition path, the pattern

shifts its shape rapidly from a wave-like shape (Fig.S11D) to an ellipse shape (Fig.S10E) and finally to a spot shape (Fig.S11F). In short, the dominant modes N_{01} and N_{10} during the nucleation process lead to a rapid increase of the maximum magnitude of vegetation biomass n with nearly unchanged spatial pattern shape before the transition state at the saddle point (before the nucleation formation), while the shape alteration mostly takes place after the nucleation formation.

Moreover, we may compare the effects of different dominant modes on the nucleation process. We focus on the transition state at the saddle point $N_{01} = N_{10} = 0.94$, $N_{11} = 1.98$ (Fig.S11D), and solely adjust N_{11} with fixed $N_{01} = N_{10} = 0.94$ to create a selected set of transition states, which can be dominated by different modes. For instance, if $N_{11} = 2.28$ (Fig.S10C), Δ for N_{11} changes to be 1 so that N_{01} and N_{10} dominate the nucleation process. If $N_{11} = 1.44$ (Fig.S10D), Δ for N_{11} becomes 0 such that N_{11} dominates the nucleation process. Snapshots of vegetation patterns at these two selected transition states are illustrated in Fig.S10C and D. It is found that when N_{01} and N_{10} dominate the nucleation process, the pattern shape remains nearly stripe shape (wave-like shape) but the maximum magnitude of vegetation biomass in regions of the hexagonal lattice increases significantly (Fig.S10C) at the transition state. Conversely, when N_{11} dominates the nucleation process, the pattern shape undergoes obvious changes to an ellipse shape but the maximum magnitude of vegetation biomass increases only slightly (Fig.S10D) at the transition state. In conclusion, dominant modes N_{01} and N_{10} tend to increase the maximum magnitude of vegetation biomass n before the nucleation formation but change the pattern shape later, while the dominant mode N_{11} prefers to transform the pattern shape before the nucleation formation but increase the maximum magnitude of vegetation biomass n later.

Additionally, to verify the conclusion observed in the mode space, we perform simulations in the spatial location starting from the vegetation pattern with a stripe shape of Fig.S11A and present typical snapshots of vegetation patterns in Fig.S11G-I. The maximum magnitude of vegetation biomass increases rapidly while the pattern shape changes slowly. The pattern firstly switches from the stripe shape (Fig.S11A) to the wave-like/stripe shape (Fig.S11G), consistent with those patterns before the saddle point in the mode space (Fig.S11B-D). Then it turns to be the wave/ellipse shape (Fig.S11H), in agreement with patterns around or just past the saddle point in the mode space (Fig.S11D,E). Finally it reaches the spot shape (Fig.S11I), corresponding to patterns at the paths ending point in the mode space (Fig.S11F). Here, the vegetation pattern with spot shape in Fig.S11I is less regular than

that observed in the mode space (Fig.S11F), because the evolution of patterns in the real space would be strongly influenced by the boundary condition and other factors. All these results demonstrate that N_{01} and N_{10} are the dominant modes during the nucleation process when the ecosystem transforming from B state with the potential well located in the region $N_{01} = N_{10} = 0$, $N_{11} > 0$ to H_0 state with the well located in the region $N_{01} = N_{10} = N_{11} > 0$.

Definitions of C_1, C_2 and $\overline{C}$

In addition, to measure the time irreversibility of the system more clearly, we introduce the two-point cross-correlations forward C_1 and backward C_2 in time as well as their average differences $\overline{C}$. Due to the 3 slow-changing modes, there exists 3 pairs of two-point cross-correlations. Taking the cross-correlation between N_{01} and N_{10} as an example, functions of $C_1(N_{01}, N_{10})$ and $C_2(N_{01}, N_{10})$ can be described as:

$$C_1(\tau) = \langle N_{01}(0)N_{10}(\tau)\rangle, \tag{S37}$$

$$C_2(\tau) = \langle N_{01}(\tau)N_{10}(0)\rangle, \tag{S38}$$

which can also be written as $\sum N_{01}^i N_{10}^j P_i^{ss} P_{ij}(\tau)$, where P_i^{ss} denotes the steady-state probability at i state and $P_{ij}(\tau)$ represents the probability changing from i state to j state with time interval τ [23, 24]. The nonequilibrium steady-state probability flux can be defined as $J_{ij}^{ss} = P_i^{ss}k_{ij} - P_j^{ss}k_{ji} \approx P_i^{ss}P_{ij}(\tau) - P_j^{ss}P_{ji}(\tau)$ since $k_{ij}\tau \approx \tau P_{ij}(\tau)$ for small τ, where k_{ij} (k_{ji}) indicates the transition rate from i (j) state to j (i) state. Therefore, the difference between C_1 and C_2 can be given by [23, 24] $C_1(\tau) - C_2(\tau) = N_{01}^i N_{10}^j [P_i^{ss}P_{ij}(\tau) - P_j^{ss}P_{ji}(\tau)] = N_{01}^i N_{10}^j J_{ij}^{ss}\tau$. The average differences between C_1 and C_2 is defined as

$$\overline{C} = \sqrt{\int_0^{t_0} (C_1(\tau) - C_2(\tau))^2 d\tau / t_0}, \tag{S39}$$

which can quantify the time irreversibility of the semi-arid ecosystem.

Influences of the size effect (noise intensity)

We perform intensive simulations to understand the influences of other parameters, such as the ecosystem size, on our main results. Analyzing the structure of the diffusion matrix, $\mathbf{D}$, and the time correlation of the noise terms, reveals an inverse proportionality between

noise and the square of the ecosystem size, i.e., as the ecosystem size decreases, the noise intensity increases. The EPR, e_p, is shown as a function of β for 4 different ecosystem sizes in Fig.S12A-D, respectively. Notably, it is observed that the decrease of e_p in the H_π/B coexisting phase transforms into a plateau as the ecosystem size decreases to $k = 17$ and $L_b = 59.1$ (Fig.S12A) first, and shifts to an ascending curve without any peaks after the size decreases to $k = 13$ and $L_b = 45.2$ (Fig.S12B). Similarly, the decrease of EPR in the B/H_0 coexisting phase reaches a plateau as the ecosystem size decreases to $k = 9$ and $L_b = 31.3$ (Fig.S12C), and transforms into an ascending curve without any peaks after the size decreases to $k = 5$ and $L_b = 17.4$ (Fig.S12D). In other words, as the ecosystem size becomes smaller (effectively increasing noise), the potential landscape will become flatter, resulting in the critical transition changing from a discontinuous to a continuous process, or even vanishing altogether.

Influences of other parameters

In addition to the ecosystem size, we also varied a number of other parameters and observed results qualitatively similar to the ones in the main text. Two examples of semi-arid ecosystems, with parameters $a = 4.4$, $m = 1.8$, $\alpha = 10$ and $a = 4.8$, $m = 1.95$, $\alpha = 10$ are shown in Fig.S13 and Fig.S14, respectively. According to the phase diagram in the deterministic model without any noise (Fig.S13A and Fig.S14A), the vegetation patterns switch from gap to stripe and eventually to spot patterns, consistent with the observations in the aforementioned ecosystem. The obtained EPR dependent on β is presented in Fig.S13B and Fig.S14B in a similar manner. Although the change of e_p in the H_π/B coexisting phase is hidden, e_p still decreases in the B/H_0 coexisting phase and shows peaks at the phase boundaries, which can also be used to serve as an early warning for the desertification in the semi-arid ecosystem with given parameters. As the ecosystem size is reduced (increasing the noise intensity), the decrease of e_p in the B/H_0 coexisting phase transforms into a plateau, and eventually into an ascending curve without any peaks (Fig.S13B-D and Fig.S14B-D). These results demonstrate the generality and robustness of both the phenomena of the pattern formation and switching in semi-arid ecosystems and our analytical method. Moreover, the phase transition threshold of β from the stripe pattern to the spot one is located in different regions: around 0.18 for $a = 8.0$ (Fig.S12), around 0.1 for $a = 4.4$ (Fig.S13) and around 0.11 for $a = 4.8$ (Fig.S14). It is found that as a becomes smaller, the transition

threshold of β decreases, implying that lower water input will lead to a easier formation of the spot pattern or desertification.

---

[1] Christopher A Klausmeier. Regular and irregular patterns in semiarid vegetation. *Science*, 284(5421):1826–1828, 1999.

[2] Jost von Hardenberg, Ehud Meron, Moshe Shachak, and Yair Zarmi. Diversity of vegetation patterns and desertification. *Physical review letters*, 87(19):198101, 2001.

[3] Gui-Quan Sun, Cui-Hua Wang, Li-Li Chang, Yong-Ping Wu, Li Li, and Zhen Jin. Effects of feedback regulation on vegetation patterns in semi-arid environments. *Applied Mathematical Modelling*, 61:200–215, 2018.

[4] Wei Wu and Jin Wang. Landscape framework and global stability for stochastic reaction diffusion and general spatially extended systems with intrinsic fluctuations. *The Journal of Physical Chemistry B*, 117(42):12908–12934, 2013.

[5] Crispin W Gardiner et al. *Handbook of stochastic methods*, volume 3. springer Berlin, 1985.

[6] Michael E Peskin. *An introduction to quantum field theory*. CRC press, 2018.

[7] Claude Itzykson and Jean-Michel Drouffe. *Statistical field theory: volume 2, strong coupling, Monte Carlo methods, conformal field theory and random systems*, volume 2. Cambridge University Press, 1991.

[8] L Wettmann, M Bonny, and K Kruse. Effects of molecular noise on bistable protein distributions in rod-shaped bacteria. *Interface Focus*, 4(6):20140039, 2014.

[9] Li Xu, Denis Patterson, Simon Asher Levin, and Jin Wang. Non-equilibrium early-warning signals for critical transitions in ecological systems. *Proceedings of the National Academy of Sciences*, 120(5):e2218663120, 2023.

[10] Jin Wang, Li Xu, and Erkang Wang. Potential landscape and flux framework of nonequilibrium networks: robustness, dissipation, and coherence of biochemical oscillations. *Proc. Natl. Acad. Sci. U. S. A.*, 105(34):12271–12276, 2008.

[11] Chunhe Li and Jin Wang. Landscape and flux reveal a new global view and physical quantification of mammalian cell cycle. *Proc. Natl. Acad. Sci. U. S. A.*, 111(39):14130–14135, 2014.

[12] Xiaona Fang, Karsten Kruse, Ting Lu, and Jin Wang. Nonequilibrium physics in biology. *Rev. Mod. Phys.*, 91(4):045004, 2019.

[13] Xiakun Chu and Jin Wang. Conformational state switching and pathways of chromosome dynamics in cell cycle. *Appl. Phys. Rev.*, 7(3):031403, 2020.

[14] Jin Wang. Landscape and flux theory of non-equilibrium dynamical systems with application to biology. *Adv. Phys.*, 64(1):1–137, 2015.

[15] Jie Su, Zhiyu Cao, Jin Wang, Huijun Jiang, and Zhonghuai Hou. Dynamical and thermodynamical origins of motility-induced phase separation. *Cell Reports Physical Science*, 5(2):101817, 2024.

[16] Xiaona Fang and Jin Wang. Nonequilibrium thermodynamics in cell biology: Extending equilibrium formalism to cover living systems. *Annu. Rev. Biophys.*, 49(1), 2020.

[17] Chunhe Li and Jin Wang. Quantifying the landscape for development and cancer from a core cancer stem cell circuit. *Cancer research*, 75(13):2607–2618, 2015.

[18] Chunhe Li and Jin Wang. Quantifying the underlying landscape and paths of cancer. *Journal of The Royal Society Interface*, 11(100):20140774, 2014.

[19] Han Yan, Lei Zhao, Liang Hu, Xidi Wang, Erkang Wang, and Jin Wang. Nonequilibrium landscape theory of neural networks. *Proceedings of the National Academy of Sciences*, 110(45):E4185–E4194, 2013.

[20] Li Xu, Denis Patterson, Ann Carla Staver, Simon Asher Levin, and Jin Wang. Unifying deterministic and stochastic ecological dynamics via a landscape-flux approach. *Proceedings of the National Academy of Sciences*, 118(24):e2103779118, 2021.

[21] Udo Seifert. Stochastic thermodynamics, fluctuation theorems and molecular machines. *Rep. Prog. Phys.*, 75(12):126001, 2012.

[22] Hao Ge and Hong Qian. Physical origins of entropy production, free energy dissipation, and their mathematical representations. *Physical Review E*, 81(5):051133, 2010.

[23] Hong Qian and Elliot L Elson. Fluorescence correlation spectroscopy with high-order and dual-color correlation to probe nonequilibrium steady states. *Proceedings of the National Academy of Sciences*, 101(9):2828–2833, 2004.

[24] Kun Zhang and Jin Wang. Exploring the underlying mechanisms of the xenopus laevis embryonic cell cycle. *The Journal of Physical Chemistry B*, 122(21):5487–5499, 2018.

[25] Fabio Borgogno, P D'odorico, Francesco Laio, and Luca Ridolfi. Mathematical models of vegetation pattern formation in ecohydrology. *Reviews of geophysics*, 47(1), 2009.

[26] Christian Valentin, Jean-Marc d'Herbès, and Jean Poesen. Soil and water components of banded vegetation patterns. *Catena*, 37(1-2):1–24, 1999.

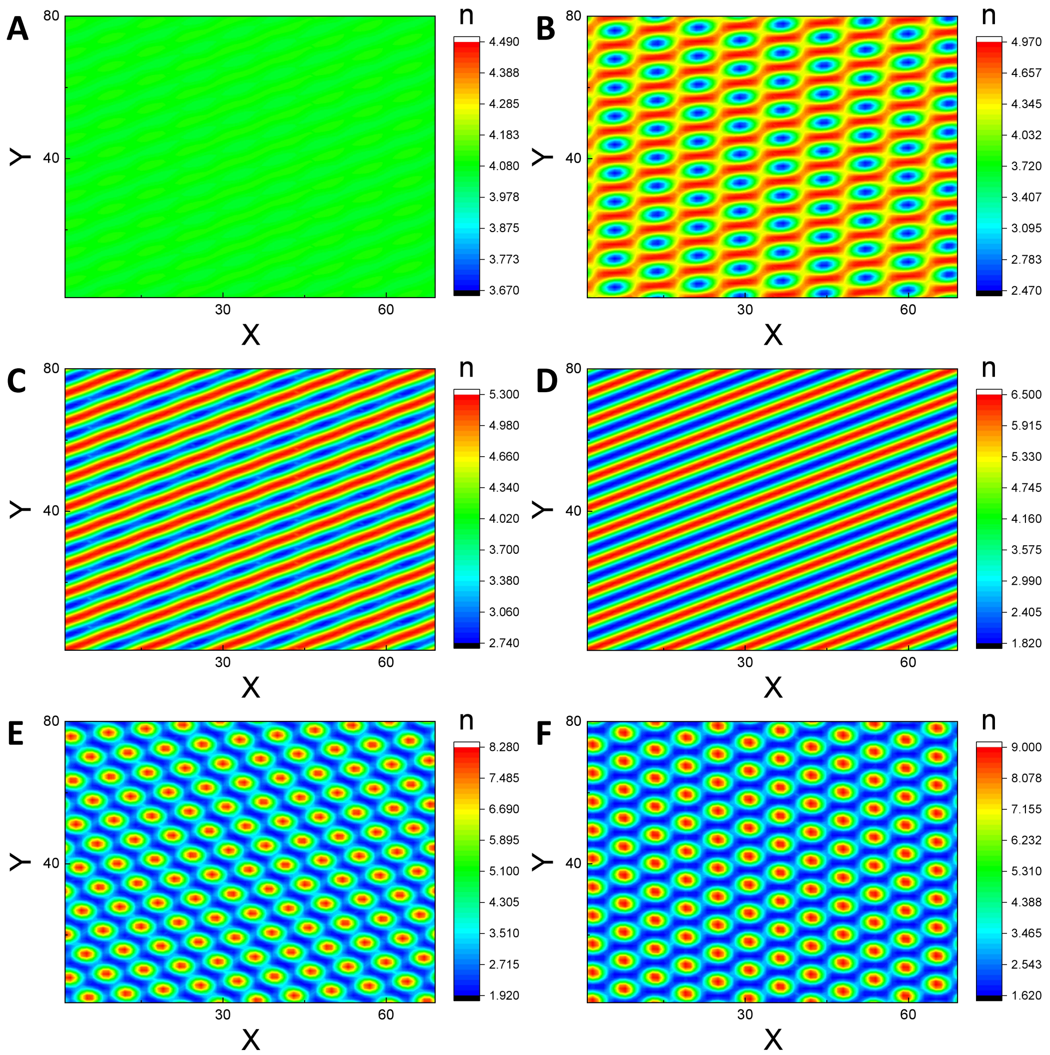

FIG. S1. **Typical snapshots of the semi-arid ecosystem from the evolution equations in the spatial location.** The parameters are $\beta = 0.001$ (**A**), 0.003 (**B**), 0.005 (**C**), 0.017 (**D**), 0.018 (**E**), 0.024 (**F**).

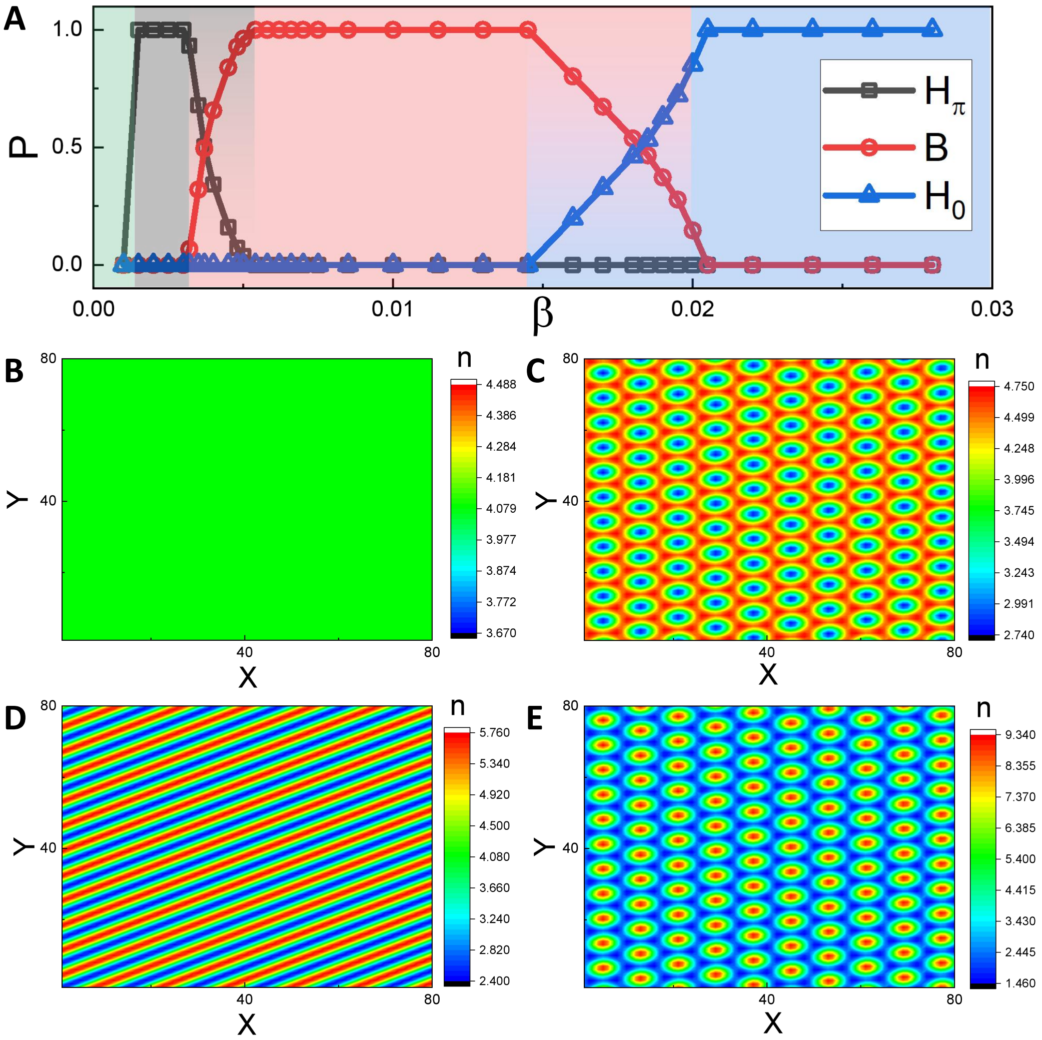

FIG. S2. **Phase diagram and typical snapshots of the semi-arid ecosystem.** (**A**). The probabilities of vegetation patterns depend on β. The black, red and blue lines represent the gap (H_π state), stripe (B state) and spot (H_0 state) patterns, respectively. Regions filled by one color represent the single pattern phase while the ones filled by two colors denote the coexisting patterns phase. Typical snapshots of the vegetation are shown in (**B**) the uniform pattern with $\beta = 0.001$, (**C**) the gap pattern with $\beta = 0.0032$, (**D**) the stripe pattern with $\beta = 0.009$, and (**E**) the spot pattern with $\beta = 0.026$.

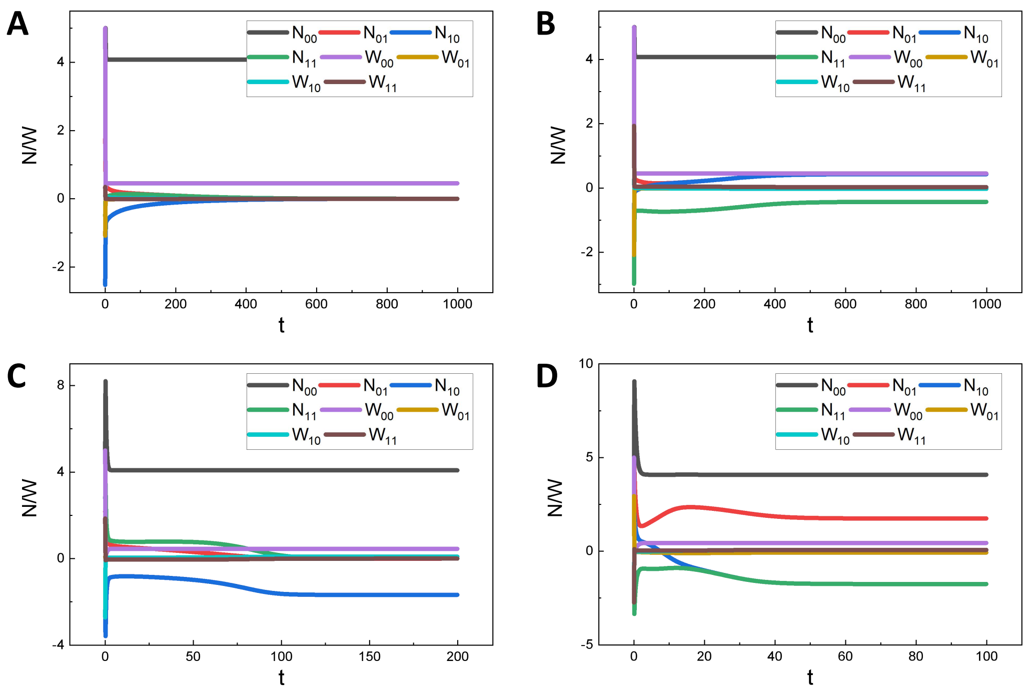

FIG. S3. **Time series of each mode.** The parameters are $\beta = 0.001$ (**A**), 0.003 (**B**), 0.09 (**C**), 0.024 (**D**).

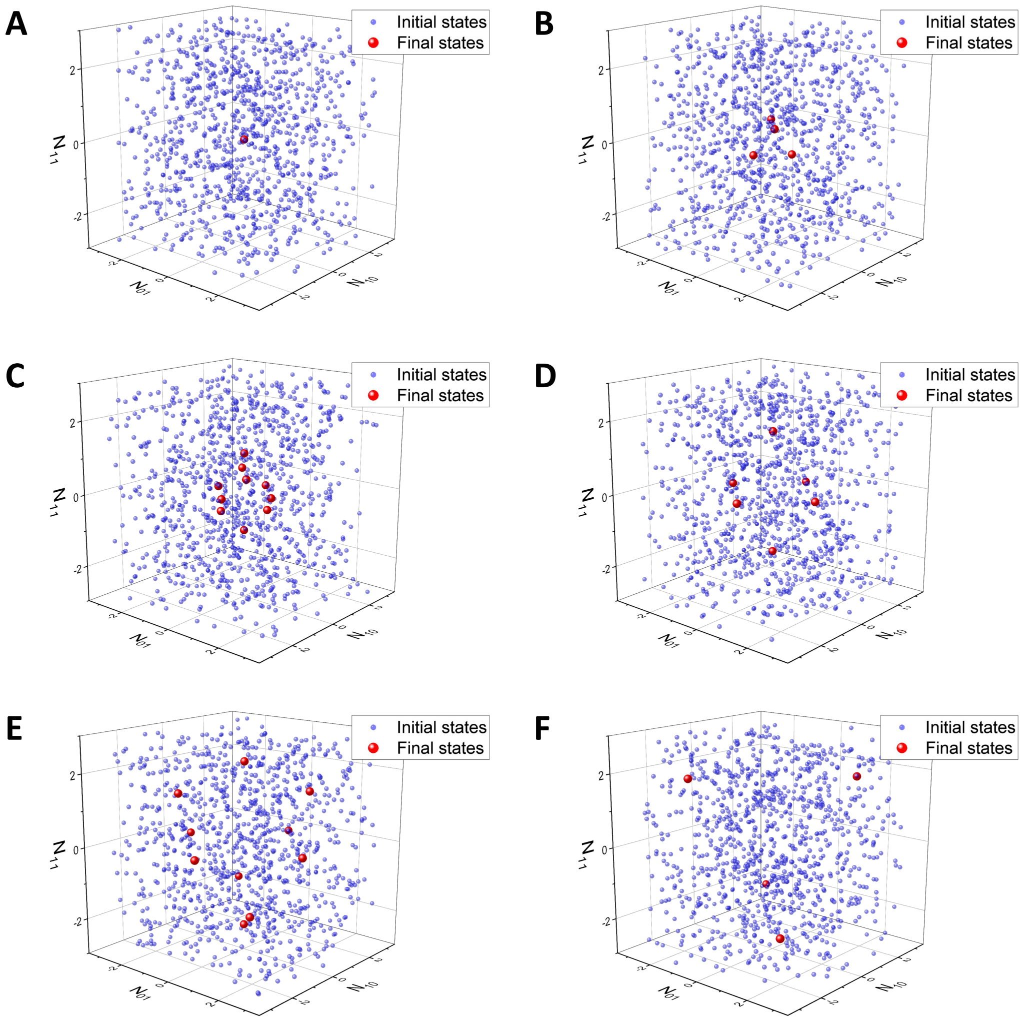

FIG. S4. **Comparison of final states in different phases.** The blue spheres are 1000 random initial states and the red spheres are final states. The parameters are $\beta = 0.001$ (**A**), 0.003 (**B**), 0.0043 (**C**), 0.009 (**D**), 0.018 (**E**), 0.026 (**F**).

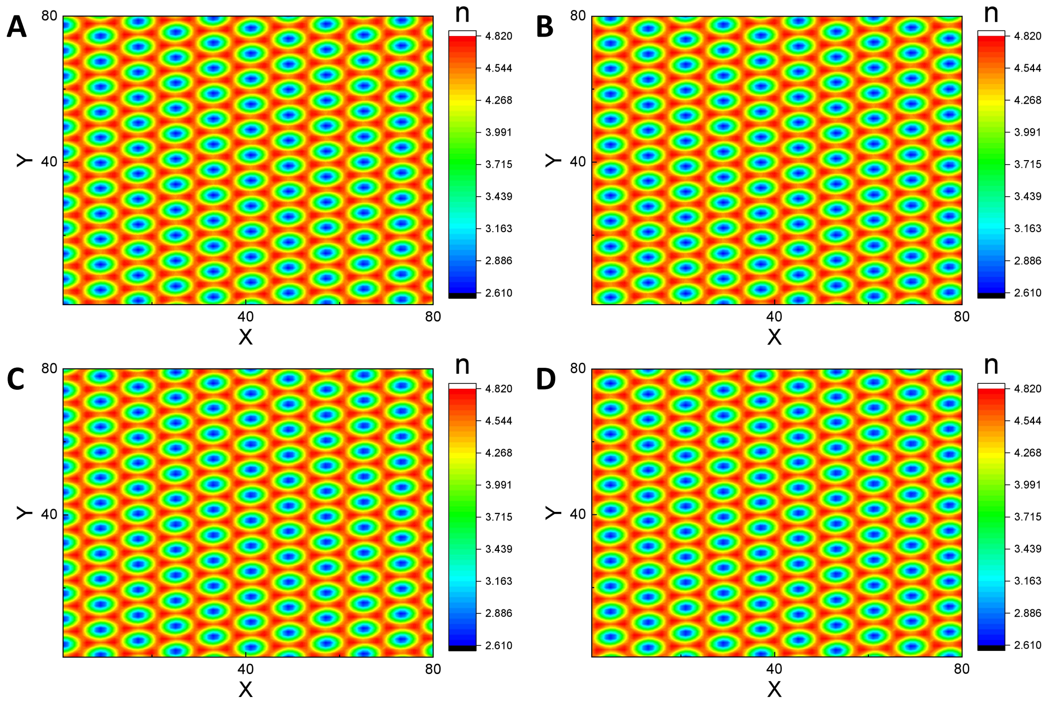

FIG. S5. **Snapshots of vegetation with gap patterns for $\beta = 0.003$.** The final states are located in (**A**) $N_{01} = N_{10} = N_{11} < 0$, (**B**) $N_{01} = -N_{10} = -N_{11} < 0$, (**C**) $-N_{01} = N_{10} = -N_{11} < 0$ and (**D**) $-N_{01} = -N_{10} = N_{11} < 0$.

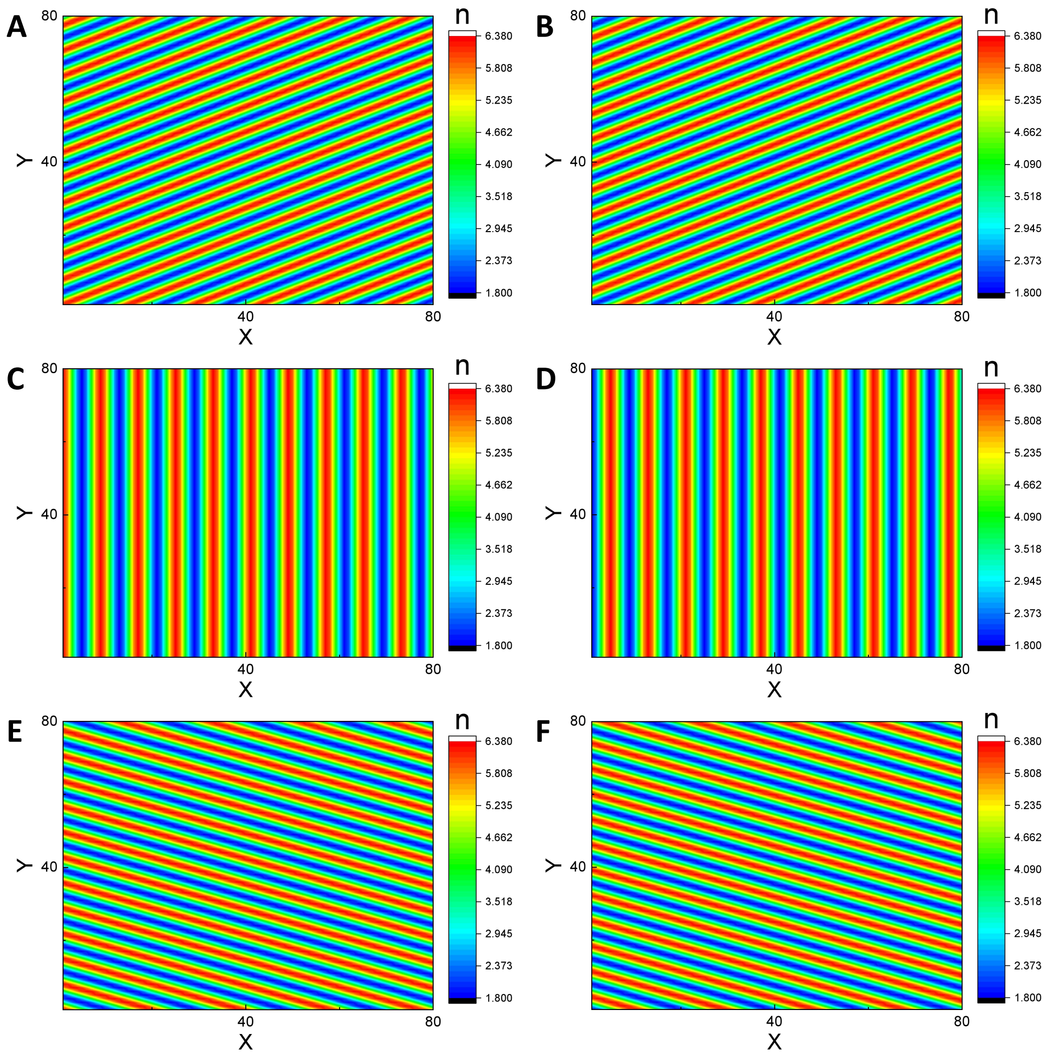

FIG. S6. **Snapshots of vegetation with stripe patterns for $\beta = 0.009$.** The final states are located in (**A**) $N_{01} > 0$, $N_{10} = N_{11} = 0$, (**B**) $N_{01} < 0$, $N_{10} = N_{11} = 0$, (**C**) $N_{10} > 0$, $N_{01} = N_{11} = 0$, (**D**) $N_{10} < 0$, $N_{01} = N_{11} = 0$, (**E**) $N_{11} > 0$, $N_{01} = N_{10} = 0$ and (**F**) $N_{11} < 0$, $N_{01} = N_{10} = 0$.

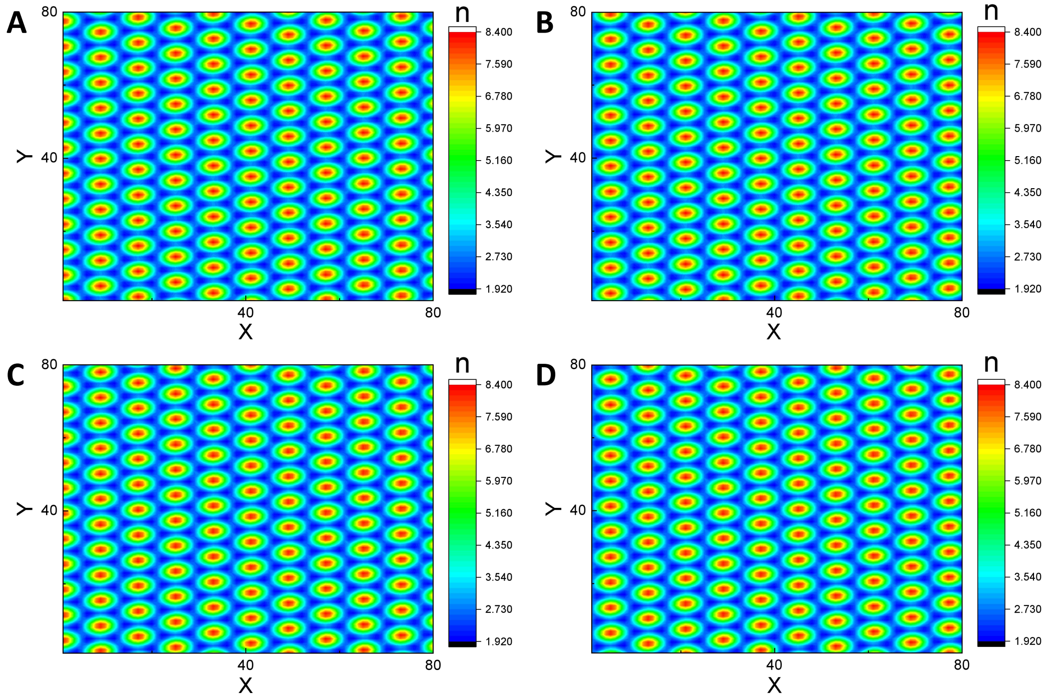

FIG. S7. **Snapshots of vegetation with spot patterns for $\beta = 0.026$.** The final states are located in (**A**) $N_{01} = N_{10} = N_{11} > 0$, (**B**) $N_{01} = -N_{10} = -N_{11} > 0$, (**C**) $-N_{01} = N_{10} = -N_{11} > 0$ and (**D**) $-N_{01} = -N_{10} = N_{11} > 0$.

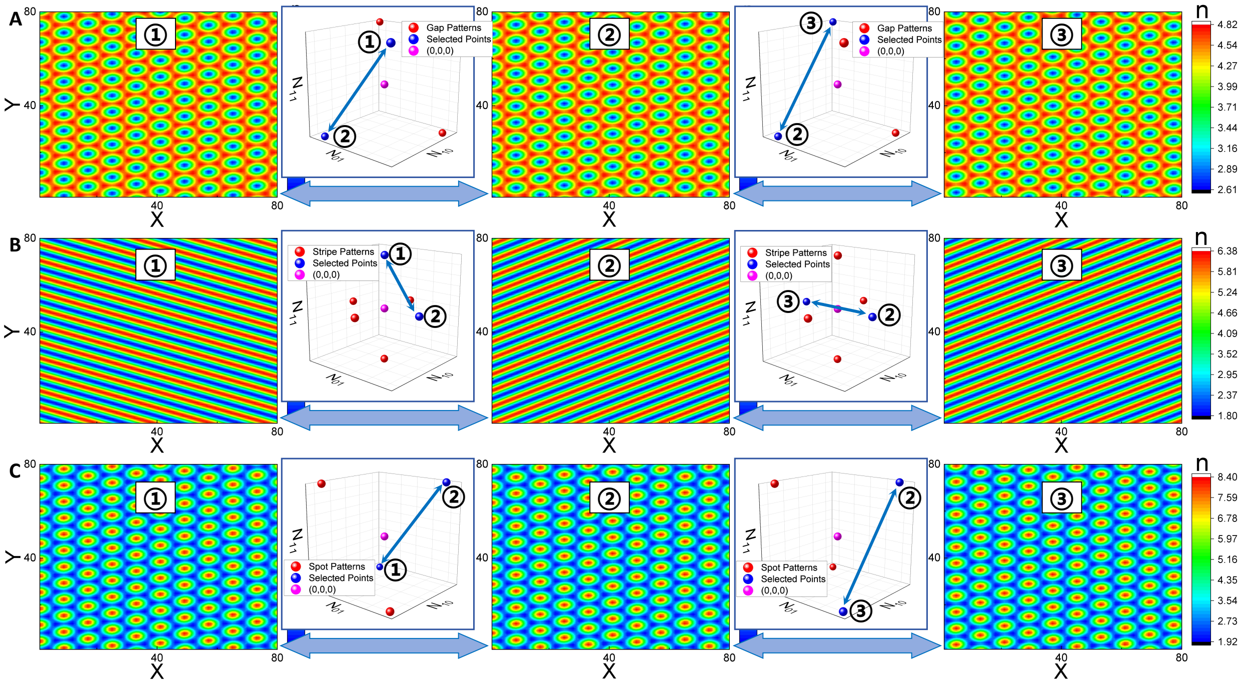

FIG. S8. **Schematic diagram that links mode amplitudes to spatial configurations.** (**A**), (**B**), and (**C**) are respectively the gap, stripe and spot patterns, including 3 patterns with different orientations or phase shifts and 2 schematic diagrams exhibiting the corresponding modes' positions. The spatial pattern and its corresponding state in the mode space (the blue point) are marked with the same number.

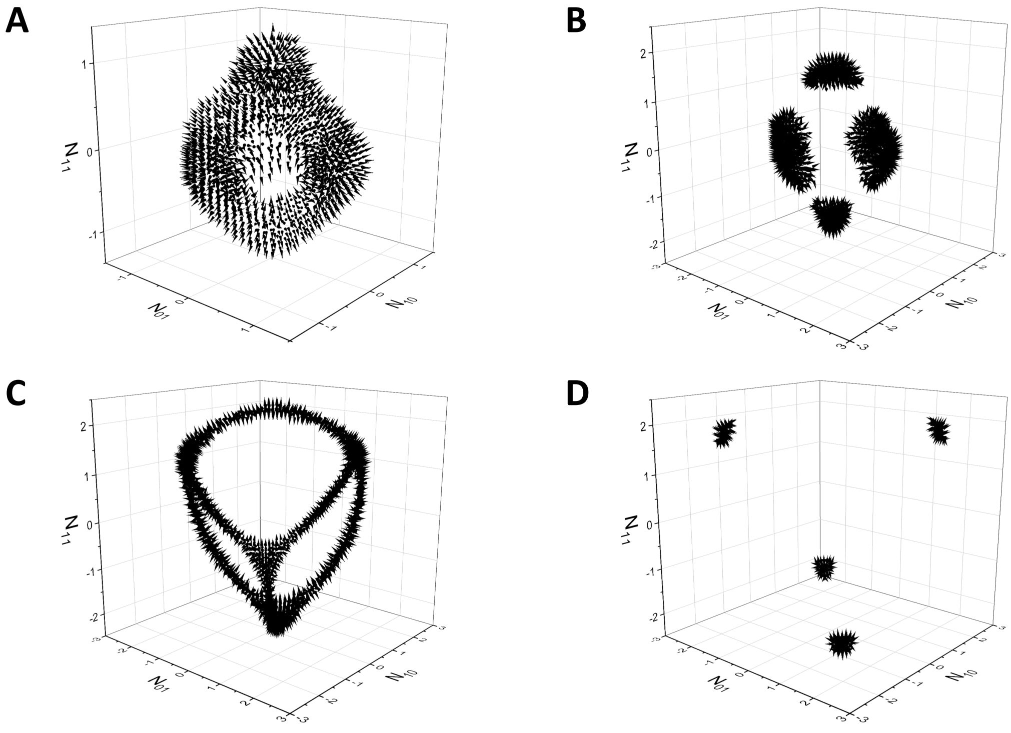

FIG. S9. **Nonequilibrium flux field in the 3-dimensional mode space.** The parameters are $\beta = 0.004$ (**A**), 0.009 (**B**), 0.018 (**C**), 0.026 (**D**).

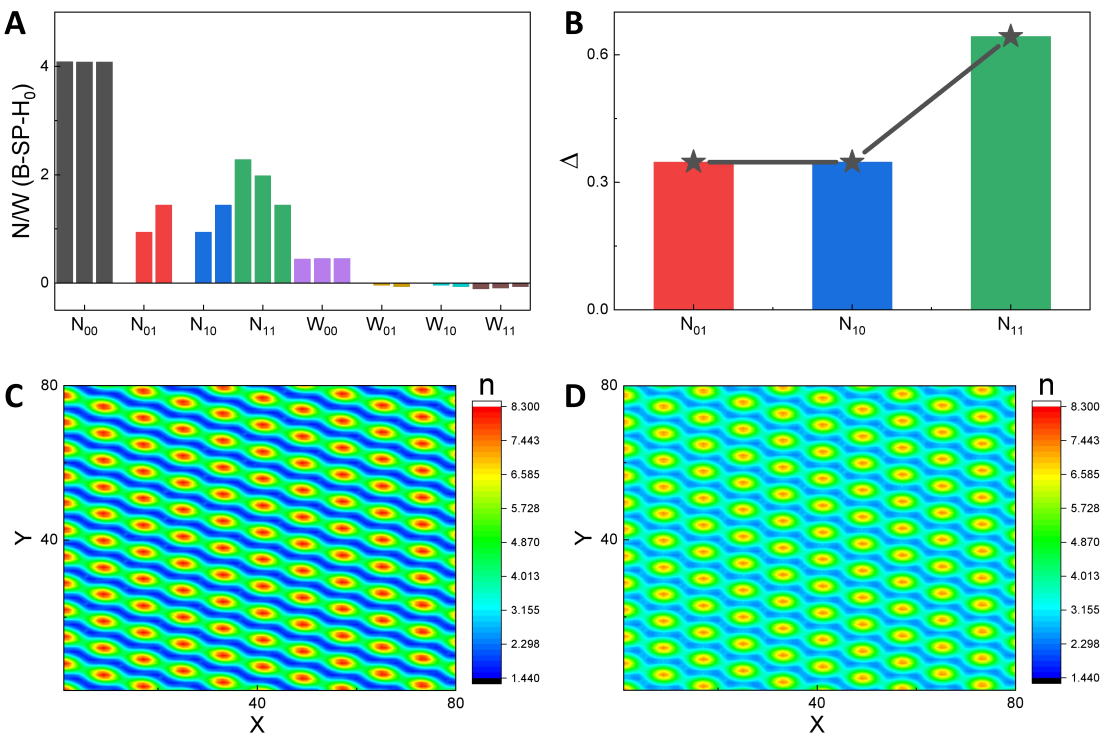

FIG. S10. **The dominant modes in the nucleation process for the semi-arid ecosystem.** (**A**) The comparison of each mode at the beginning, the ending and the saddle points of the transition path switching from B to H_0 state for $\beta = 0.018$ (the black line in Fig.4C of the main text). (**B**) The histogram of Δ dependent on N_{01}, N_{10} and N_{11} corresponding to (**A**). (**C**,**D**) The comparison of spatial vegetation patterns at selected transition states dominated by different modes in the nucleation process with (**C**) $N_{01} = N_{10} = 0.94$, $N_{11} = 2.28$ (N_{01} and N_{10} dominate the nucleation process) and (**D**) $N_{01} = N_{10} = 0.94$, $N_{11} = 1.44$ (N_{11} dominates the nucleation process).

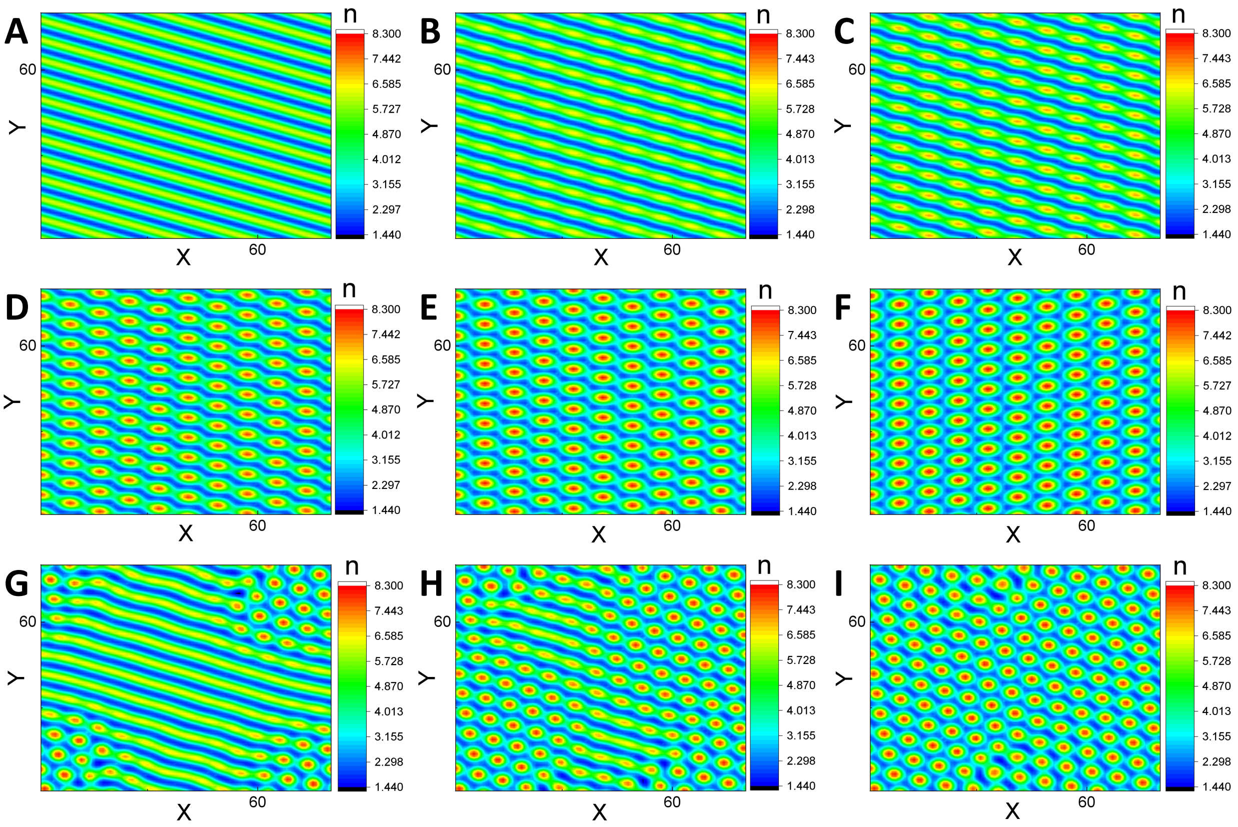

FIG. S11. **Snapshots of vegetation patterns switching from the stripe pattern to the spot pattern for** $\beta = 0.018$ **in the mode space and the real space.** (**A-F**) Vegetation patterns switching in the mode space from the transition path in Fig.4C of the main text (the black line) with (**A**) $N_{01} = N_{10} = 0$, $N_{11} = 2.28$, (**B**) $N_{01} = N_{10} = 0.2$, $N_{11} = 2.24$, (**C**) $N_{01} = N_{10} = 0.5$, $N_{11} = 2.17$, (**D**) $N_{01} = N_{10} = 0.94$, $N_{11} = 1.98$, (**E**) $N_{01} = N_{10} = 1.21$, $N_{11} = 1.76$ and (**F**) $N_{01} = N_{10} = N_{11} = 1.44$. (**G-I**) Typical snapshots for vegetation patterns evolving in the real space for (**G**) 100000 steps, (**H**) 200000 steps and (**I**) 1000000 steps starting from the initial state of the stripe pattern in (**A**). These two types of pattern switching both demonstrate that N_{01} and N_{10} dominate the nucleation process.

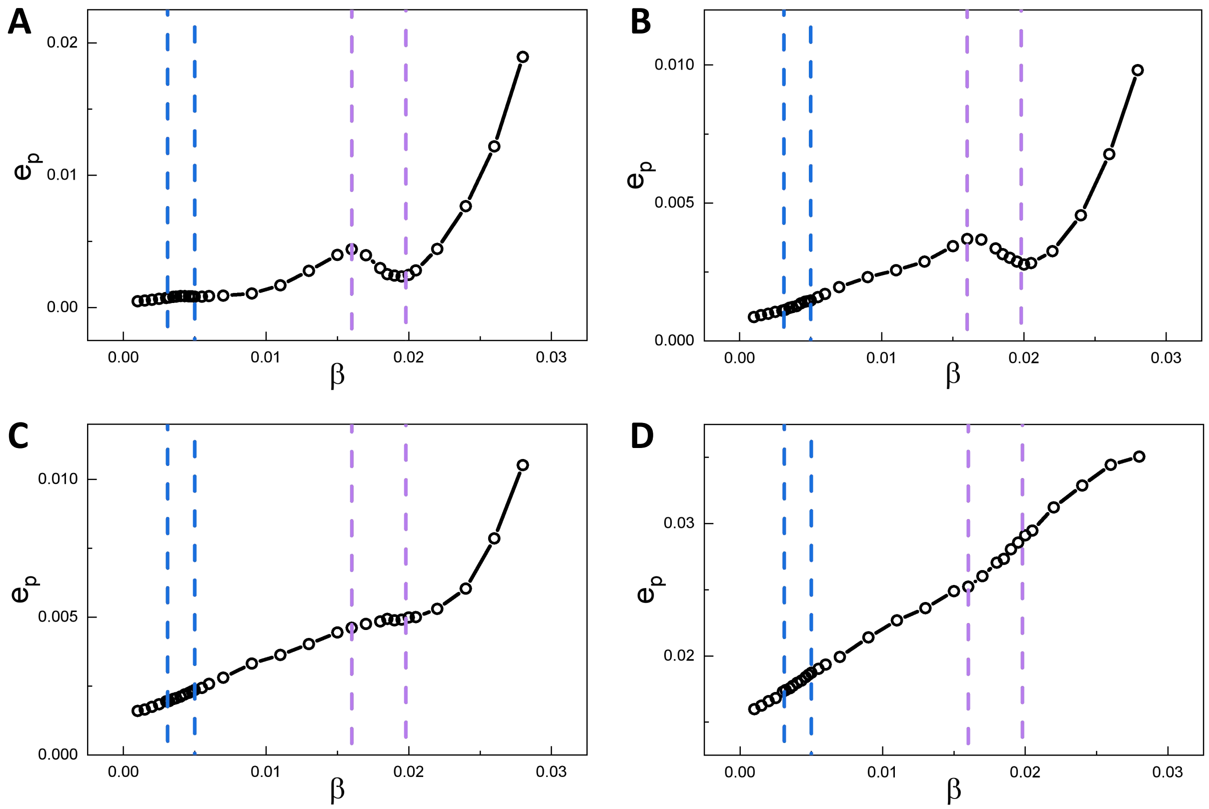

FIG. S12. **The influence of the ecosystem size (the noise intensity).** Dependence of the entropy production rate e_p on β with (**A**) $k = 17$, $L_b = 59.1$, (**B**) $k = 13$, $L_b = 45.2$, (**C**) $k = 9$, $L_b = 31.3$ and (**D**) $k = 5$, $L_b = 17.4$. The blue and purple dashed lines represent the phase boundaries between the single phases and the coexisting phases.

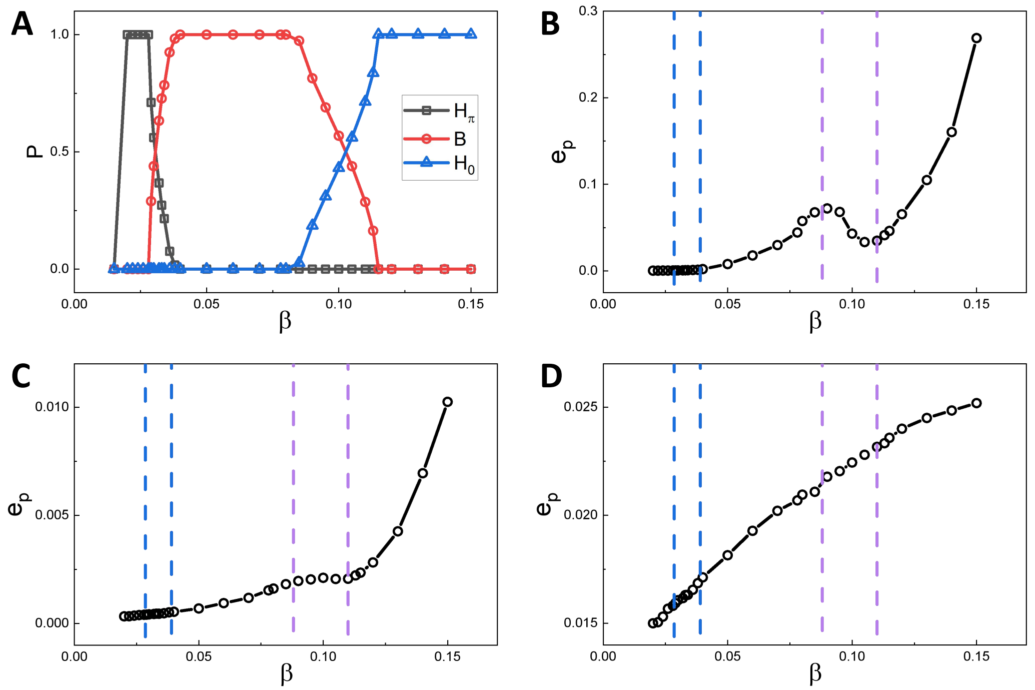

FIG. S13. **Phase diagram and the influence of the ecosystem size with parameters $a = 4.4$, $m = 1.8$ and $\alpha = 10$.** (**A**) The probabilities of vegetation patterns depend on β. The black, red and blue lines represent the gap H_π, stripe B and spot H_0 patterns, respectively. Dependence of the entropy production rate e_p on β with (**B**) $k = 34$, $L_b = 120$, (**C**) $k = 15$, $L_b = 52.9$ and (**D**) $k = 6$, $L_b = 21.2$.

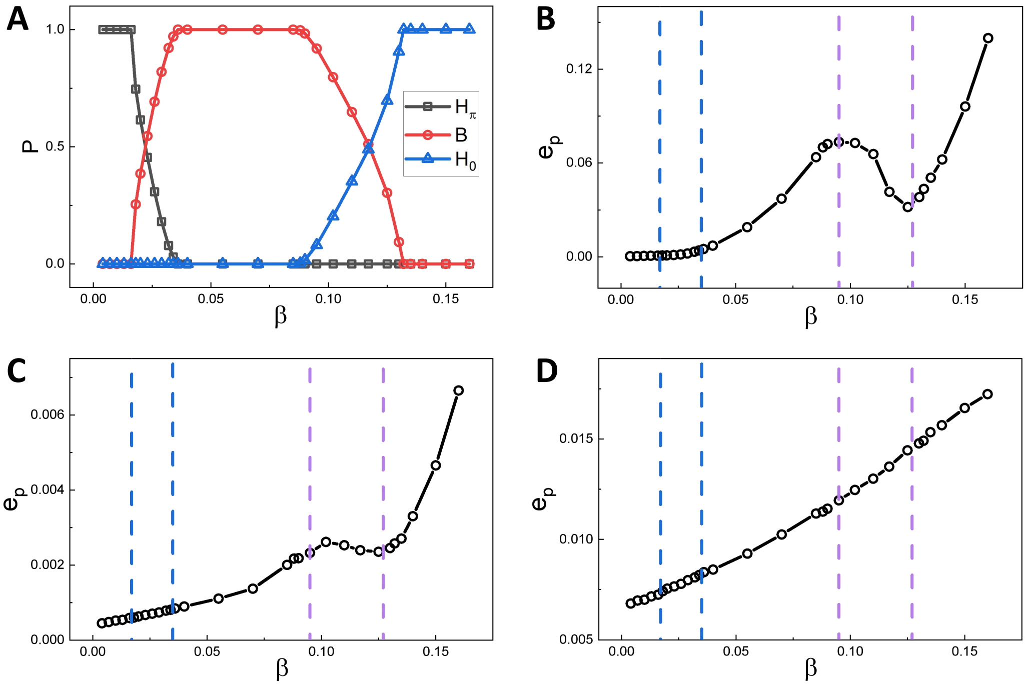

FIG. S14. **Phase diagram and the influence of the ecosystem size with parameters $a = 4.8$, $m = 1.95$ and $\alpha = 10$.** (**A**) The probabilities of vegetation patterns depend on β. The black, red and blue lines represent the gap H_π, stripe B and spot H_0 patterns, respectively. Dependence of the entropy production rate e_p on β with (**B**) $k = 34$, $L_b = 120$, (**C**) $k = 12$, $L_b = 42.4$ and (**D**) $k = 6$, $L_b = 21.2$.

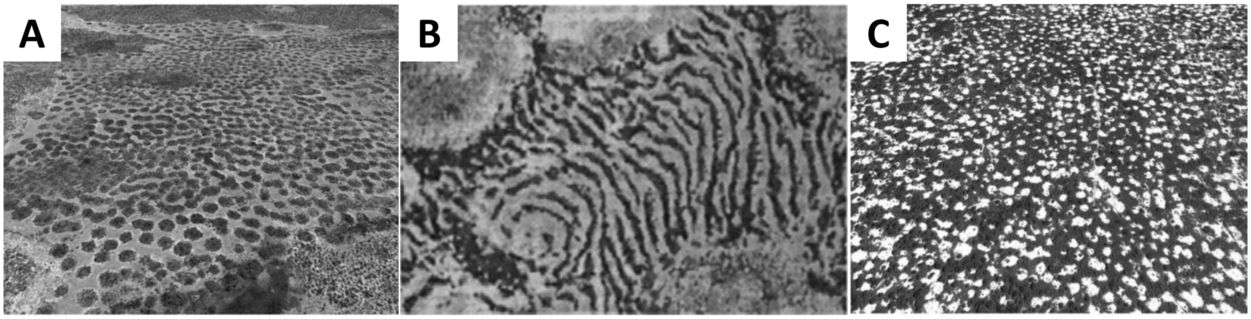

FIG. S15. **Aerial photographs of nearly periodic vegetation patterns in nature.** (**A**) A spot pattern in Zambia, reproduced with permission[25]. (**B**) A stripe pattern in Niger, reproduced with permission [26]. (**C**) A gap ("fairy circle") pattern in Senegal, reproduced with permission[25]. (A,C) Copyright 2009, John Wiley and Sons. (B) Copyright 1999, Elsevier.
